# Targeting KIF20A: a new frontier in cancer treatment revealed by multi-omics analysis

**DOI:** 10.3389/fimmu.2026.1744899

**Published:** 2026-01-21

**Authors:** Jie Zhang, Guanghao Li, Chunling Qi, Mingshan Yang, Wei Guo

**Affiliations:** 1 Shandong Provincial Key Laboratory of Precision Oncology, Shandong Cancer Hospital and Institute, Jinan, China; 2 Department of Laboratory Medicine, The Affiliated Taian City Central Hospital of Qingdao University, Taian, China; 3 Department of Urology, Shandong Cancer Hospital and Institute, Jinan, China; 4 Department of Ultrasound Medicine, Shandong Cancer Hospital and Institute, Jinan, China

**Keywords:** immunotherapy, KIF20A, multi-omics, pan-cancer, prognostic biomarker

## Abstract

**Background:**

Kinesin family member 20A (KIF20A), a microtubule-dependent motor protein of the Kinesin superfamily, is involved in cell division and organelle transport. Its expression is dysregulated in various cancers and is closely related to tumor metastasis and patient prognosis. However, its specific functions in different tumor types and the potential as an anticancer target have not been fully elucidated, and a systematic pan-cancer analysis is lacking.

**Methods:**

This study integrated multiple cancer database resources and systematically analyzed the multi-omics alterations of KIF20A in different cancers using R software, including gene expression, genomic variation, methylation status, biological pathways, and clinical value. In addition, we evaluated the regulatory role and immunotherapy potential of KIF20A in the tumor microenvironment through various bioinformatics algorithms. Finally, we explored the impact of KIF20A on the biological behaviors of Kidney Renal Clear Cell Carcinoma (KIRC) cells through *in vitro* and *in vivo* experiments.

**Results:**

KIF20A is localized in the nucleus and participates in the cell cycle process, serving as a core gene for tumor cell growth. It undergoes copy number alterations in various tumors, and its high expression is closely associated with clinical progression, poor prognosis, and activation of classical oncogenic pathways in multiple cancers. Mechanistically, aberrant epigenetic modifications and mutations in hallmark pathways are significant reasons for the dysregulated expression of KIF20A. Furthermore, the expression of KIF20A correlates with immune cell infiltration and the expression of immune checkpoint molecules, impacting the efficacy of immunotherapy in various cancers. *In vitro* experiments have confirmed that interfering with KIF20A expression can effectively inhibit the proliferation, migration, and invasion of KIRC cells. Furthermore, *in vivo* experimental results indicate that interfering with KIF20A can inhibit tumor growth in nude mice.

**Conclusion:**

To our knowledge, this is the first study to reveal the role of KIF20A in tumorigenesis and development from a pan-cancer multi-omics perspective, providing solid theoretical and experimental evidence for KIF20A as a potential anti-cancer therapeutic target.

## Introduction

Cancer remains a leading cause of mortality worldwide, with an estimated 20 million new cases and 9.7 million deaths reported in 2022 (1). Although advances in imaging and liquid biopsy have improved cancer detection rates (2, 3), the early occult nature and high metastatic potential of tumors often result in diagnosis at advanced stages (4, 5). Current treatments—including surgery, radiotherapy, chemotherapy, and targeted therapy—all face limitations. Surgery and radiotherapy are constrained by tumor location (6, 7); chemotherapy induces significant toxicity and recurrence (8), and targeted therapy is effective only in patients with specific mutations and is prone to drug resistance (9, 10). Although immune checkpoint blockade (ICB) has provided significant survival benefits in some cancers, immune tolerance leads to resistance in many patients and may trigger autoimmune reactions (11, 12). To address these challenges, pan-cancer research based on high-throughput sequencing has emerged (13, 14).

KIF20A, a member of the kinesin superfamily, is located on chromosome 5q31.2 and is also known as MKLP2 and RAB6KIFL. It encodes a molecular motor protein associated with microtubule movement, with a molecular weight of approximately 100 kDa (15). KIF20A participates in various key processes within the cell by binding to microtubules and moving with the energy produced by ATP hydrolysis, including cell division, organelle transport, and signal transduction. Particularly during mitosis, it is involved in the positioning of the dividing cell nucleus and the correct setup of the intracellular division apparatus, ensuring orderly and efficient cell division (16, 17). Numerous studies have shown that KIF20A is significantly overexpressed in various cancers such as breast cancer (18), lung cancer (19), liver cancer (20), and bladder cancer (21), and is associated with increased tumor invasiveness, disease progression, and poor clinical prognosis. For instance, in colorectal cancer and gliomas, KIF20A interacts with the JAK-STAT3 pathway, promoting tumor cell growth and metastasis (22, 23). Additionally, in ovarian clear cell carcinoma, immunohistochemical (IHC) analysis has revealed that overexpression of KIF20A can significantly promote tumor cell proliferation (24). Studies have also found that in hepatocellular carcinoma (HCC), the accumulation of KIF20A not only promotes the proliferation and tumorigenic potential of pathological liver cells but also enhances the tumor’s resistance to chemotherapy (20). Although KIF20A has been the focus of attention in various cancers, no studies have yet elucidated its role in the development and progression of tumors from a pan-cancer perspective.

In this study, we explored the expression, genomic alterations, clinical significance, and immunological value of KIF20A across various cancer types using public databases and multiple bioinformatics algorithms. Our results indicate that KIF20A is upregulated in multiple tumor types, including at both the mRNA and protein levels. High expression of KIF20A accelerates clinical malignant progression through the activation of cell cycle-related processes and oncogenic pathways and is closely associated with poor prognosis. Furthermore, KIF20A expression is closely related to genomic and immune status and affects the efficacy of immunotherapy. Finally, validation in external cohorts and cell experiments confirmed the oncogenic role of KIF20A in KIRC. In conclusion, KIF20A is a potential therapeutic target.

## Materials and methods

### Multi-omics analysis of cell lines

We obtained immunofluorescence staining images of cell lines (A-431, U-251MG, and U2OS) from the HPA database to observe the subcellular localization of KIF20A. Sequencing data for U2OS FUCCI cells used in cell cycle analysis (GSE146773) were obtained from the Gene Expression Omnibus (GEO) database. Gene expression levels were converted to z-scores using the scale function, and outliers greater than 3 or less than -3 were removed. Subsequently, standardized RNA expression profiles were plotted as functions of cell cycle phase over simulated cell cycle time. Preprocessed multi-omics data from tumor cells were obtained from the Genomics of Drug Sensitivity in Cancer (GDSC) website. Gene expression data from the Human Protein Atlas (HPA) database and copy number variation data from the Cancer Cell Line Encyclopedia (CCLE) database were used for validation. Plots were generated using basic graphics functions in R and the ggplot2 package. Finally, we downloaded genome-wide CRISPR screening data from the DepMap database to assess gene genetic dependencies. The CERES algorithm was employed to calculate KIF20A dependency scores in cancer cells, where negative scores indicate cell growth inhibition or death following gene knockout.

### Single-cell sequencing analysis

We obtained pan-cancer single-cell expression profiles annotated with cell type information from the Tumour Immune Single−Cell Hub (TISCH) database. Using the R package pheatmap, we constructed heatmaps to visualize the pan-cancer single-cell expression landscape of KIF20A. Subsequently, we calculated the average expression level of KIF20A in each single-cell dataset and the proportion of specific cells expressing KIF20A in each dataset. Spearman correlation analysis was performed to assess the relationship between these two metrics, visualized using lollipop plots. We performed dimensionality reduction on the single-cell data using Uniform Manifold Approximation and Projection (UMAP). Different cell types were visualized as a two-dimensional heatmap based on their unique expression patterns. Finally, all cells were classified into KIF20A-positive and -negative groups based on expression status, and the proportion of each cell type within the positive and negative groups was calculated separately.

### Multi-omics analysis of the TCGA pan-cancer cohort

Multi-omics data from The Cancer Genome Atlas (TCGA) pan-cancer cohort were obtained from the UCSC xena database, encompassing gene expression, DNA methylation, copy number variations, somatic mutations, and clinical information. Gene expression data for normal human tissues were sourced from the GTEx database. Based on tissue origin, we merged these with TCGA transcriptomic data using the R package Combat and performed inter-group differential analysis with the R package Limma. Based on sample type information and KIF20A expression data, we employed the R package pROC to evaluate KIF20A’s diagnostic value across pan-cancer types. Clinical ORR data were obtained from prior studies. We merged KIF20A expression levels from the TCGA pan-cancer cohort with ORR data, then performed Pearson correlation analysis to investigate their relationship (25). Extract each patient’s distinct survival endpoints (OS, DSS, DFI, and PFI) and survival status information from clinical data. Perform univariate Cox regression analysis and Kaplan-Meier survival analysis using the R package survival. Based on pathological staging and histological grade information in the clinical data, compare KIF20A expression differences between groups using Wilcoxon Rank Sum Tests. Methylation sites for KIF20A were extracted from DNA methylation data. Based on sample type information, Wilcoxon Rank Sum Tests were performed to assess differences in KIF20A methylation status between normal and tumor tissues. Pearson correlation tests were conducted to evaluate the relationship between KIF20A expression levels and methylation beta values. Genomic State Score (Aneuploidy Score, Homologous Recombination Defects, Fraction Altered, Number of Segments, Intratumor Heterogeneity, Nonsilent Mutation Rate, Silent Mutation Rate, CTA Score, Indel Neoantigens and SNV Neoantigens) and TMB data were obtained from previous studies. KIF20A expression was integrated with these data based on TCGA sample names, followed by correlation analysis and differential analysis (26). Extract mutation information for 12 molecules (NOTCH1, TP53, WNT1, MAPK1, EGFR, BRAF, KRAS, TGFBR1, PIK3CA, MYC, YAP1, and CDKN1A) from SNV somatic mutation data. The SNV mutation frequency (percentage) for each gene coding region was calculated as: number of mutated samples/number of cancer samples. Heatmaps were generated using the R package pheatmap. Additionally, based on whether mutations occurred in the 12 molecules, we used the Wilcoxon signed-rank test to observe changes in KIF20A expression (up- or down-regulated) in the mutated group compared to the wild-type group.

### Proteomics analysis of a pan-cancer cohort

Protein expression data and corresponding clinical information for the pan-cancer cohort were obtained from the Proteomic Data Commons (PDC) database (https://pdc.cancer.gov/pdc/). Missing values in the matrix were imputed using the R package “Impute.” Based on sample and cancer type information, we employed Wilcoxon Rank Sum Tests to explore differences in KIF20A protein expression between normal and tumor tissues across various cancers. Additionally, we validated KIF20A protein expression using the Cancer Proteome and Phosphoproteome Atlas (CPPA) online tool. Immunohistochemical scores and images from normal and tumor tissues of different organ origins were obtained from the HPA database. The R package ggplot2 was used to plot percentage bar charts illustrating the proportion of different KIF20A staining scores in tumor tissues.

Tumor function-associated proteins were obtained from The Cancer Proteome Atlas (TCPA) database, and Spearman correlations between KIF20A expression and specific functional protein content were calculated using the cor.test function. Protein interactions were analyzed using the Compartmentalized Protein−Protein Interaction (ComPPI) online database. For proteins localized to the cell nucleus, GO-BP enrichment analysis was performed using the R package clusterProfiler.

### Pathway enrichment analysis

In the TCGA pan-cancer cohort, samples were divided into high-expression and low-expression groups based on the median KIF20A expression level for each cancer type. Differential analysis was performed using the limma package to obtain the log2FC for each gene. All genes were ranked by log2FC. Based on hallmark gene sets obtained from Molecular Signatures Database (MSigDB) and Kyoto Encyclopedia of Genes and Genomes (KEGG) metabolic gene sets, Gene set enrichment analysis was performed using the Gene Set Enrichment Analysis (GSEA) function in the clusterProfiler package. Enrichment scores (ES) were calculated for each gene set, followed by significance testing and multiple hypothesis correction. Results were visualized using bubble plots. Nine tumor cell functional gene sets (Proliferation, Apoptosis, Cell Cycle, EMT, Metastasis, Invasion, DNA damage, DNA repair, and Stemness) were retrieved from the Cancer Single−cell State Atlas (CancerSEA) database. The Gene Set Variation Analysis (GSVA) R package performed gene set variation analysis to obtain nine functional scores for each sample. Pearson correlation tests explored the relationship between KIF20A expression and these nine functional scores. The correlation between KIF20A at the protein level and ten classic oncogenic pathways (Apoptosis, Cell Cycle, DNA Damage, EMT, Hormone AR, Hormone ER, PI3K AKT, RAS MAPK, RTK, and TSC mTOR) was performed using the Gene Set Cancer Analysis (GSCA) online tool.

### Immunological analysis

The TCGA pan-cancer immuno-subtype data were obtained from previous studies. Tumor samples were divided into high- and low-expression groups based on the median KIF20A expression level. The proportion of each subtype within each group was calculated, and significance was assessed using the chi-square test (26). Cancer Immune Periodicity Scores were obtained from the TIP database, while tumor microenvironment scores from two algorithms (Xcell and Estimate) were retrieved from the Sangerbox website. Pearson correlation analysis was performed to explore the relationship between KIF20A expression and these scores across different cancers. Tumor lymphocyte infiltration images were obtained from the TilMaps database, and pan-cancer immune cell association analysis was performed using the TIMER 2.0 website. Immune checkpoint genes were obtained from the Tumor−Immune System Interaction (TISIDB) database. We selected eight clinically relevant immune checkpoints (CD274, CTLA4, HAVCR2, LAG3, PDCD1, PDCD1LG2, SIGLEC15, and TIGIT). Within the TCGA pan-cancer cohort, Pearson correlation analysis was performed between each checkpoint gene and KIF20A using the cor.test function, with heatmaps visualized via the ComplexHeatmap package. Immune biomarker scores for the TCGA pan-cancer cohort were obtained from the Tumor Immune Dysfunction and Exclusion (TIDE) database. Samples were stratified into high/low score groups based on the median biomarker score. Bar charts illustrate the proportion of patients with high/low KIF20A expression across different groups. Assessment of KIF20A’s impact on immunotherapy efficacy in real-world cohorts was performed using the BEST website.

### Verification of KIRC external queues

Spatial transcriptomics analysis was performed using the SpatialTME website. Spearman correlation analysis was employed to explore the relationship between KIF20A expression and cellular content, visualized using the linkET package. Expression profiles and clinical data from the KIRC dataset (GSE167573) were obtained from the GEO database, with probe conversion and normalization of expression data completed using Sanggerbox. Wilcoxon Rank Sum Tests were employed to compare intergroup differences, while the R package survival was used to explore survival analysis in KIF20A high/low expression groups. Additionally, we validated KIF20A expression in normal, tumor, and metastatic samples using renal carcinoma chip cohorts from the TNMplot website. Finally, we assessed the role of KIF20A in renal carcinoma immunotherapy cohorts (CheckMate025) via the IMPACT website.

### Cell culture

786-o and ACHN human clear cell renal cell carcinoma lines were procured from the Cell Bank of the Chinese Academy of Sciences in Shanghai. The cell lines are free of mycoplasma contamination and have been identified by STR analysis. We’ve grown the 786-o cells in RPMI 1640 medium from Gibco in Carlsbad, California, while the ACHN cells were nurtured in DMEM from the same company. Both cell lines were fortified with 10% fetal bovine serum from Gibco, plus 1% antibiotics—a blend of penicillin and streptomycin at a concentration of 100 units per milliliter. The culturing environment was maintained at 37 degrees Celsius with a 5% CO2 atmosphere.

### *In vitro* proliferation, migration and wound-healing assays

Cell Proliferation assay: KIRC cells were cultured in 96-well plates at 2,000 cells/well under standard conditions (37°C, 5% CO_2_). Daily CCK-8 assays (Sparkjade, China) measured cell proliferation over five days, with absorbance readings taken at 450 nm following one-hour incubation.

Cell wound-healing assay: Cells were seeded in 6-well plates, incubated to 95% confluence, wounded, and treated with serum-free medium to assess migration over time via microscopy and ImageJ analysis.

Cell migration and invasion assay: To assess cell migration, serum-starved KIRC cells (6×10^4^) were seeded in transwell inserts coated with 25% matrigel. The lower chamber contained 10% FBS medium. Following 8-24hour incubation, non-migrated cells were removed, and remaining cells were fixed, stained with crystal violet, and quantified microscopically using ImageJ.

### Tumor xenograft model

In a tumor xenograft model, 12 five-week-old female BALB/c nude mice were randomly assigned to an experimental group and a control group. The experimental group received a subcutaneous injection of the ACHN cell line with KIF20A knockdown into the hind limb, while the control group was injected with the control shRNA cell line. The cell concentration for each injection was 1×10^6^ cells per mouse. We kept a close eye on tumor development and calculated tumor volume using the standard formula *V = (L × W²)/*2, where *L* represents the longest tumor dimension and *W* indicates the shortest. Once measurements were complete, the tumors were surgically removed, photographed, weighed, preserved, and stained for further analysis.

### Statistical analysis

Statistical analysis for this study was performed using GraphPad Prism software (version 9) and R software (version 4.2.1). A two-tailed P-value < 0.05 was considered statistically significant. The levels of significance were indicated as * P < 0.05, ** P < 0.01, *** P < 0.001, **** *P <* 0.0001. The correlation of gene expression levels between two datasets was assessed using Spearman rank correlation test. Inter-group gene expression differences were compared using the Wilcoxon rank-sum test. Survival differences among different groups were analyzed using the Log-rank test. For the selection of prognostic factors, univariate Cox proportional hazards regression model was first used to identify potential factors, followed by multivariate Cox proportional hazards regression model to validate independent prognostic factors. For different analysis modules, we applied differentiated multiple comparison strategies: analyses involving a large number of simultaneous tests were corrected for false discovery rate (FDR) using the Benjamini-Hochberg method (with an FDR < 0.05 as the significance threshold). Exploratory correlation analyses with fewer tests were controlled for false positives by setting a stringent raw *P*-value threshold of < 0.05. For *in vitro* functional experiments, each experimental condition was set with three independent replicate wells. The experimental data were summarized from at least three independent experimental repetitions before statistical analysis.

## Result

### The localization, multi-omics characteristics and functions of KIF20A in cancer cells

**Figure 1:**
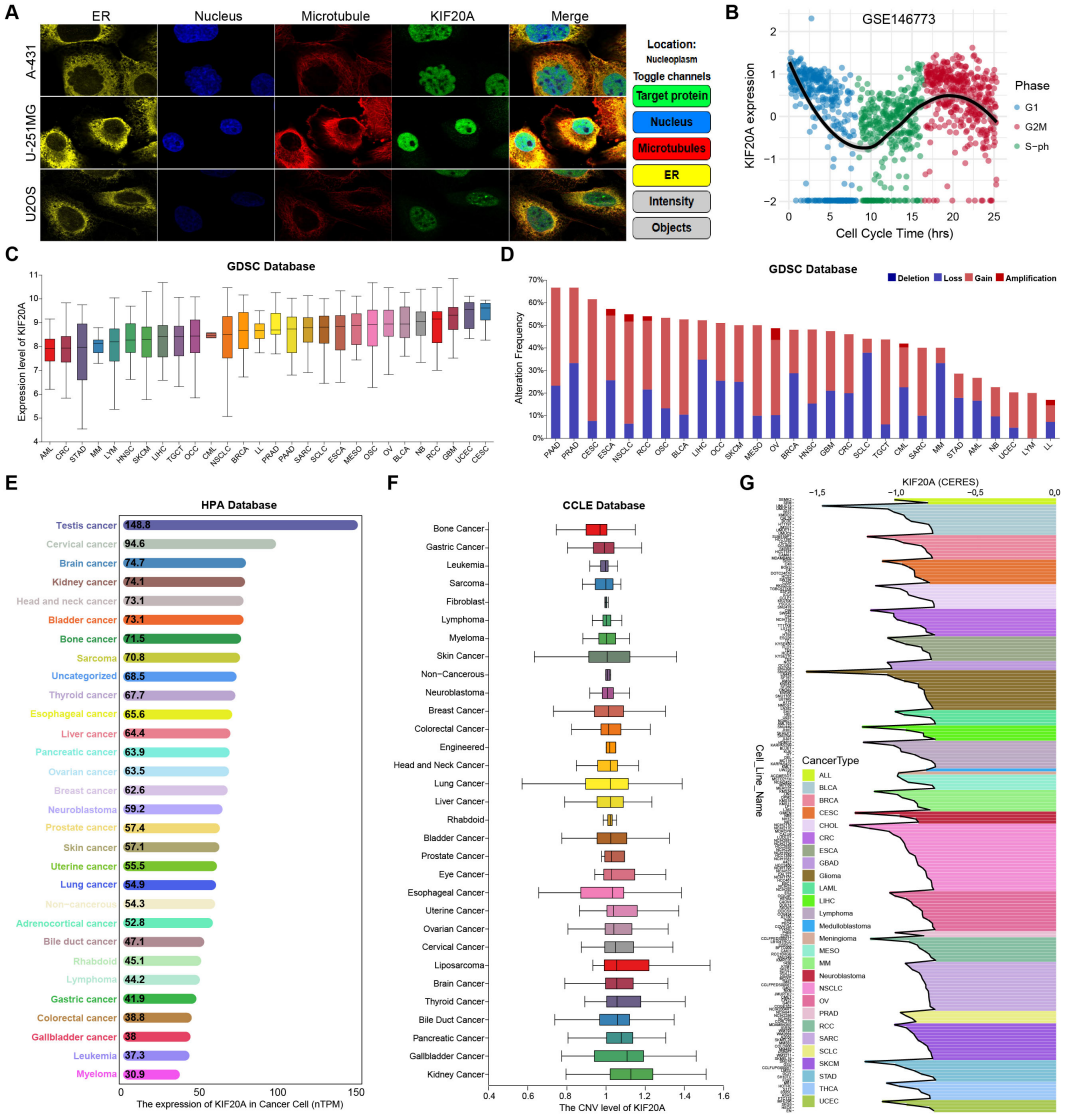
Subcellular localization, cell cycle expression, and functional dependencies of KIF20A in tumor cells. **(A)** Immunofluorescence staining revealed subcellular localization of KIF20A in A-431, U-251MG and U2OS cells. **(B)** The scatterplot showed the mRNA expression levels of KIF20A in U2OS FUCCI cells at different cell cycle phases. **(C)** mRNA expression levels of KIF20A in different tumor cell lines in GDSC database. **(D)** The change frequency of KIF20A gene copy number in different tumor cells in GDSC database. **(E)** mRNA expression levels of KIF20A in different tumor cell lines in HPA database. **(F)** CNV levels of KIF20A in different tumor cells in CCLE database. **(G)** The knock-out effect of KIF20A in different tumor cells in the DepMap database revealed its genetic dependence.

To clarify the subcellular localization of KIF20A in cells, we retrieved the immunofluorescence staining images of cell lines derived from three different tumors in the HPA database, including skin cancer (A-431), glioma (U-251MG), and osteosarcoma (U2OS). The results demonstrated that KIF20A was mainly localized in the nucleus in all three cell lines (**Figure 1A**). Furthermore, based on the FUCCI fluorescent marker-based cell cycle dataset (GSE146773), we explored the impact of KIF20A on cell cycle progression at the single-cell level. The results indicated that in U2OS cells, the mRNA expression of KIF20A varied with the cell cycle phase, with significantly higher expression levels in the G2/M phase compared to the G1 and S phases (**Figure 1B**). In the GDSC database, we explored the mRNA expression and genomic copy number alterations of KIF20A in tumor cells. The results showed that the expression and copy number alterations of KIF20A exhibited significant heterogeneity across different tumors. At the mRNA level, KIF20A was highly expressed in cells such as Cervical squamous cell carcinoma and endocervical adenocarcinoma (CESC), Uterine Corpus Endometrial Carcinoma (UCEC), Glioblastoma (GBM), and Renal Cell Carcinoma (RCC), while it was expressed at lower levels in cells such as Acute Myeloid Leukemia (AML), Colorectal Cancer (CRC), and Stomach Adenocarcinoma (STAD) (**Figure 1C**). At the copy number level, KIF20A showed a high frequency of alterations in cells such as Pancreatic adenocarcinoma (PAAD), Prostate adenocarcinoma (PRAD), CESC, Esophageal carcinoma (ESCA), Non−small cell lung cancer (NSCLC), and RCC (**Figure 1D**). Among these, the amplification frequency was highest in CESC and the deletion frequency was highest in Small Cell Lung Cancer (SCLC). Additionally, we performed expression (HPA database) and copy number (CCLE database) analyses of KIF20A in additional tumor cell datasets and obtained consistent results (**Figures 1E, F**). Finally, based on the whole-genome CRISPR-Cas9 screening data obtained from the DepMap database, we assessed the knockout effect of KIF20A in tumor cells, which reflects the survival dependency of tumor cells on KIF20A. The results showed that, despite heterogeneity across different cell lines and tissues, the dependency score (CERES) of KIF20A in different tumors was negative, indicating that the growth of tumor cells was inhibited after KIF20A knockout (**Figure 1G**). The above results indicate that KIF20A is localized in the nucleus and participates in cell cycle progression. It undergoes multi-omics alterations in tumor cells and is a key target for regulating tumor cell growth and proliferation.

### The expression pattern of KIF20A in the tumor microenvironment exhibits cell-subtype heterogeneity

**Figure 2:**
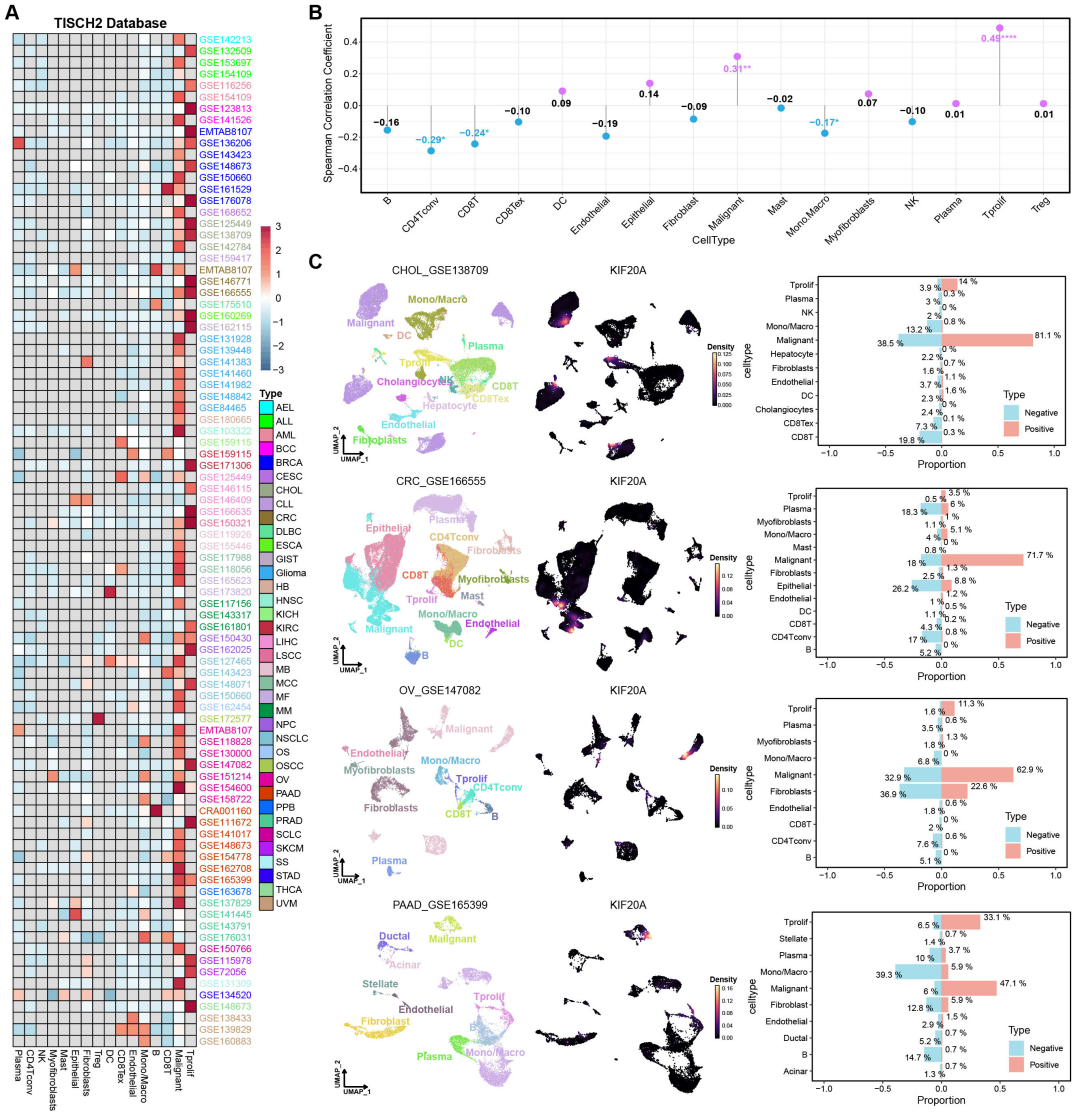
Single-cell analysis revealed the heterogeneity of KIF20A expression in the tumor microenvironment. **(A)** The pan-cancer single-cell expression landscape of KIF20A in the TISCH2 database. **(B)** Correlation analysis of KIF20A expression and specific cell content in pan-cancer microenvironment. **(C)** Expression of KIF20A at single-cell resolution in cholangiocarcinoma, colorectal cancer, ovarian cancer, and pancreatic cancer.

To delve deeper into the heterogeneity of KIF20A within the tumor microenvironment, we conducted a comprehensive single-cell analysis using the TISCH2 database. We obtained expression profiles of KIF20A from 88 single-cell datasets spanning 37 types of cancer, and through a heatmap, we illustrated the pan-cancer expression landscape of KIF20A at single-cell resolution. The results revealed substantial heterogeneity in KIF20A expression across different cell types (**Figure 2A**). To elucidate the relationship between KIF20A expression and the abundance of specific cell types, we performed a correlation analysis between the average expression levels of KIF20A and the content of different cell types. Spearman correlation analysis indicated that KIF20A expression was significantly positively correlated with malignant cells and proliferating T cells, indicating that KIF20A is highly expressed in these cells (**Figure 2B**). To validate these findings, we explored the expression of KIF20A in single-cell datasets of tumors from four different tissue origins, including cholangiocarcinoma (CHOL_GSE138709), colorectal cancer (CRC_GSE166555), ovarian cancer (OV_GSE147082), and pancreatic cancer (PAAD_GSE165399). The UMAP diagrams after dimensionality reduction and the inter-group difference analysis showed relatively consistent results among different datasets, that is, KIF20A was highly expressed in malignant cells and proliferating cells, while hardly expressed in other cell types. Additionally, the percentage bar graphs showed that the proportion of malignant cells and T-proliferating cells in the KIF20A-positive group was much higher than in the KIF20A-negative group (**Figure 2C**; **Supplementary Figure S1**). These results suggest that the expression pattern of KIF20A in tumor tissues is cell-subtype-specific, with high expression in malignant cells potentially playing a crucial role in driving tumor progression.

### Pan-cancer multi-omics analysis reveals the diagnostic, therapeutic, and prognostic values of KIF20A in human cancers

**Figure 3:**
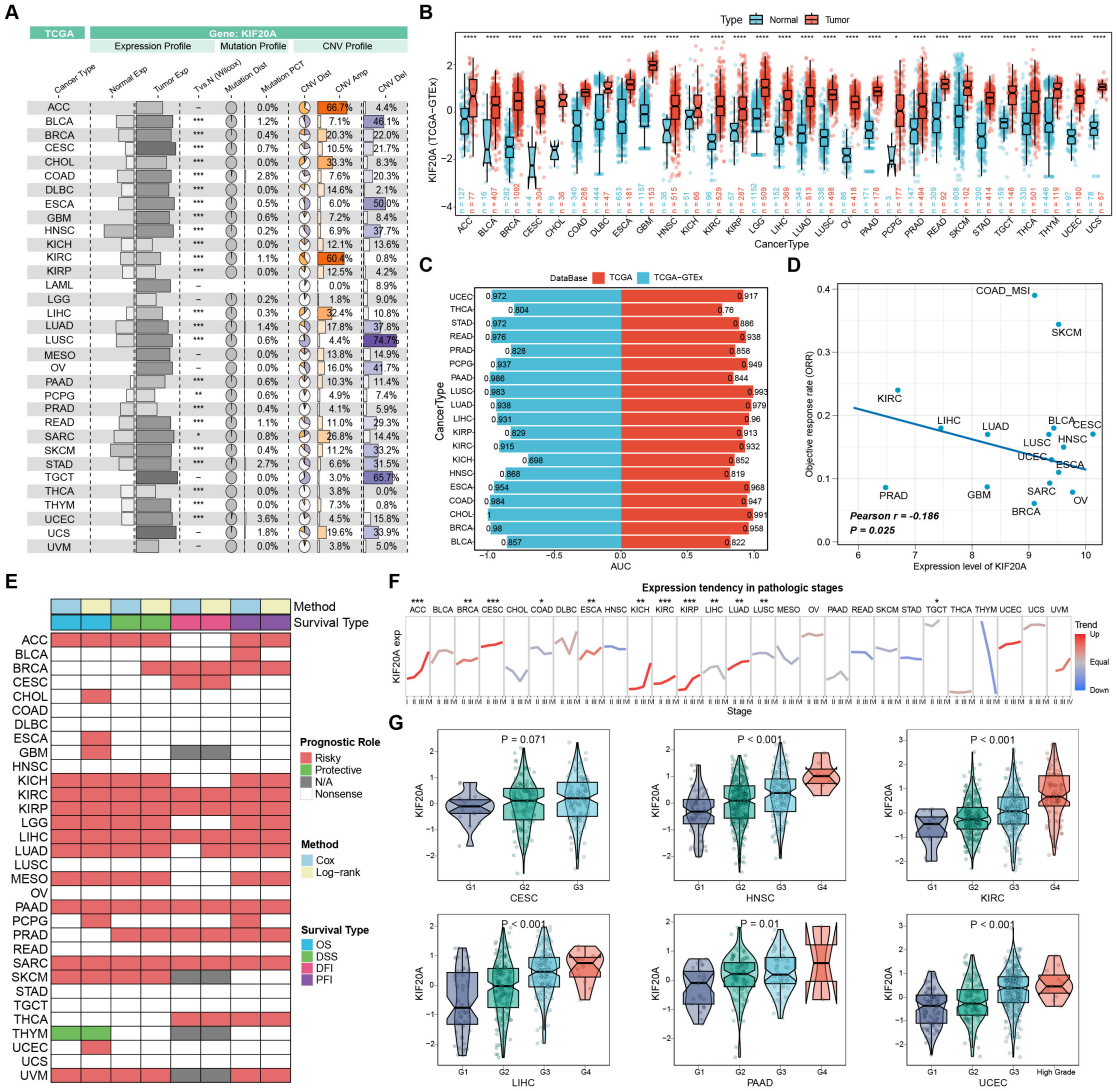
Multi-omics characteristics, diagnostic value, and clinical significance of KIF20A across pan-cancer cohorts in TCGA. **(A)** Overview of KIF20A expression, mutations, and copy number variations across multiple cancer types in TCGA. **(B)** The expression difference of KIF20A between normal and tumor tissues was analyzed by integrating TCGA and GTEx samples. **(C)** ROC analysis revealed the diagnostic value of KIF20A in pan-cancer. **(D)** The scatter plot shows the correlation between KIF20A expression levels and clinical objective response rates in 16 cancers. **(E)** Survival analysis of KIF20A in TCGA pan-cancer cohort. **(F)** Expression trend of KIF20A in different pathological stages. **(G)** Expression differences of KIF20A in different tumor grades. * P < 0.05, *** P < 0.001, **** P < 0.0001.

In order to comprehensively analyze the multi-omics characteristics and clinical value of KIF20A in tumor tissues, we analyzed data from the TCGA pan-cancer cohort. The overview map shows the multi-omics features of KIF20A in pan-cancers. We found that there were significant expression differences of KIF20A in most types of tumor tissues compared with adjacent non-tumor tissues. In addition, genomic analysis revealed that although the mutation frequency of KIF20A was low, it showed varying degrees of copy number amplification and deletion in different cancer types. For example, KIF20A had the highest frequency of copy number amplification in ACC, while it had the highest frequency of copy number deletion in Lung Squamous Cell Carcinoma (LUSC) (**Figure 3A**). Subsequently, based on the barcodes of TCGA samples, we explored the expression changes of KIF20A in a pan-cancer cohort using paired analysis. The results showed that compared to adjacent normal tissues, the mRNA expression of KIF20A was significantly upregulated in 17 types of tumor tissues, including Bladder Urothelial Carcinoma (BLCA), Breast Invasive Carcinoma (BRCA), Colon Adenocarcinoma (COAD), and ESCA, among others (**Supplementary Figure S2A**). To further clarify the expression differences of KIF20A between normal and tumor tissues, we combined the normal tissues from the GTEx database according to the organ origin with the adjacent non-tumor tissues from the TCGA. The results of differential analysis showed that the expression of KIF20A was significantly up-regulated in tumor tissues of all cancer types compared with normal tissues (**Figure 3B**). Subsequently, we evaluated the diagnostic value of KIF20A. Diagnostic ROC analysis showed that the AUC values of KIF20A were greater than 0.75 in 19 cancer types, suggesting that it is an accurate diagnostic marker for these tumors (**Figure 3C**). Additionally, we evaluated the association between KIF20A expression and clinical response in 16 types of cancer. Correlation analysis revealed a significant negative correlation between KIF20A expression levels and clinical objective response rates. Furthermore, high KIF20A expression was significantly associated with first-line treatment failure across multiple solid tumors, suggesting that elevated KIF20A expression may correlate with reduced treatment benefit and tumor progression (**Figure 3D**; **Supplementary Figure S2B**). To gain a deeper understanding of the clinical significance of KIF20A, we evaluated the prognostic value of KIF20A in the pan-cancer cohort using two algorithms (COX regression and logistic regression) and four survival endpoints (OS, DSS, DFI, and PFI). Survival analysis results showed relatively consistent results among different algorithms and survival endpoints, that is, high-expression of KIF20A was associated with poor prognosis in most tumors and was a high-risk gene for patient survival (**Figure 3E**). Additionally, we conducted a separate analysis for KIRC and found that KIF20A expression levels showed a significant linear positive correlation with survival risk in KIRC patients. This correlation remained an independent prognostic factor after multivariable adjustment (**Supplementary Figure S2C**). In addition, we investigated the expression trend of KIF20A at different pathological stages. The results showed that the expression of KIF20A increased with the increase of pathological stage in Adrenocortical carcinoma (ACC), BRCA, CESC, ESCA, Kidney Chromophobe (KICH), KIRC, Kidney Renal Papillary Cell Carcinoma (KIRP), and Lung Adenocarcinoma (LUAD), suggesting that high-expression of KIF20A may be closely related to the malignant progression of tumors (**Figure 3F**). Finally, we explored the correlation between the expression of KIF20A and histological grade in six types of cancers, including CESC, Head and Neck Squamous Cell Carcinoma (HNSC), KIRC, Liver Hepatocellular Carcinoma (LIHC), PAAD, and UCEC. Inter-group difference analysis showed that the expression of KIF20A gradually increased with the decrease of differentiation degree, suggesting that high-expression of KIF20A and low tumor differentiation may synergistically promote tumor progression and the formation of intratumoral heterogeneity (**Figure 3G**). The above results indicate that multi - omics alterations of KIF20A occur in human tumor tissues, and high-expression of KIF20A is closely related to poorer treatment response, clinical progression, and poor prognosis of patients, making it an accurate pan-cancer diagnostic marker.

### The upregulation of KIF20A protein expression in tumor tissues marks it as a key target for cell cycle regulation

**Figure 4:**
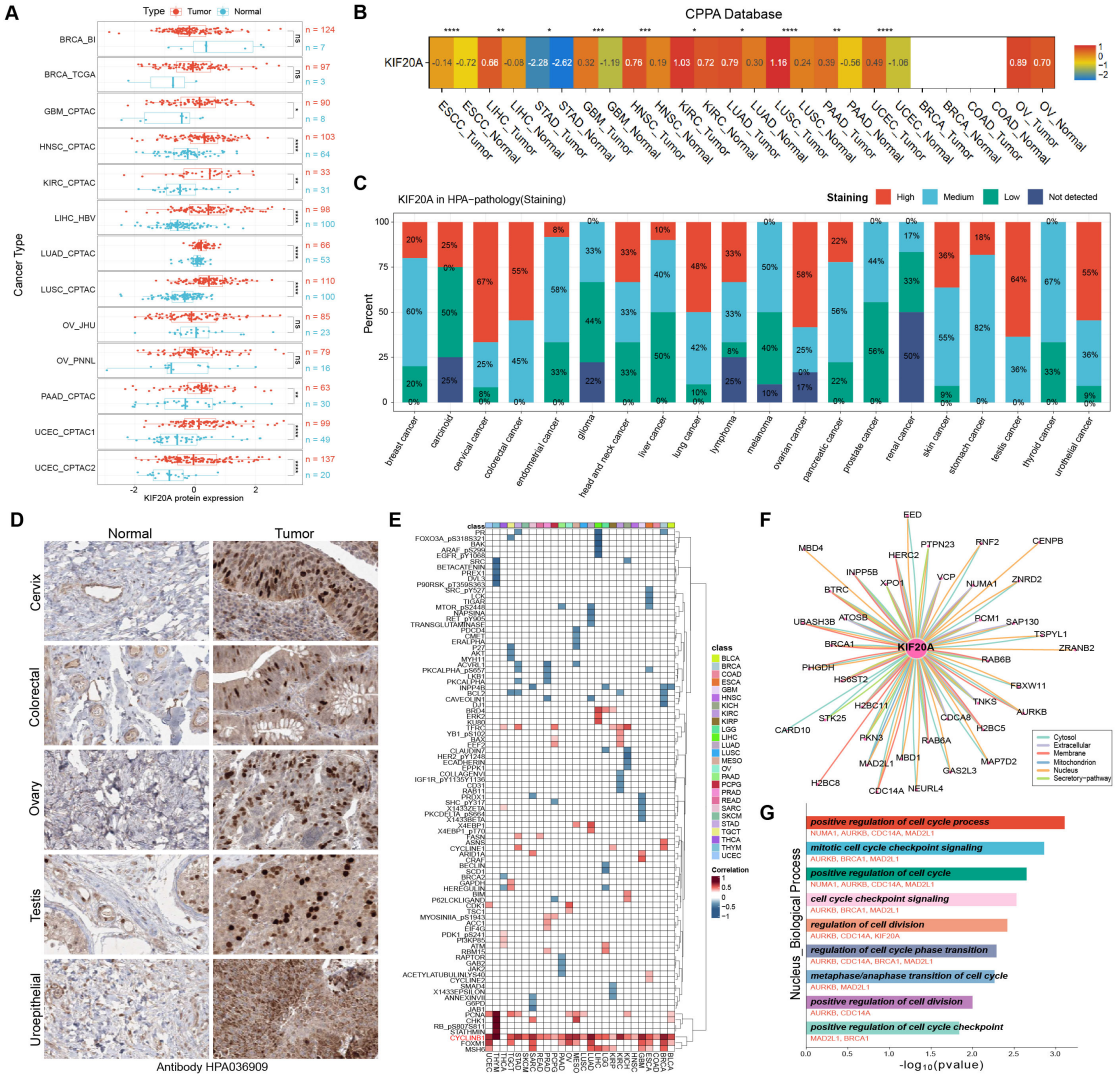
Protein expression and function analysis of KIF20A in tumor tissues. **(A)** Protein expression analysis of KIF20A in tumor and normal tissues in CPTAC database. **(B)** The CPPA online tool verified the difference in protein expression of KIF20A between tumor and normal tissues. **(C)** Immunohistochemical staining score of KIF20A protein in different types of cancer from HPA database. **(D)** Immunohistochemical staining images of KIF20A in tumor and normal tissues. **(E)** Heatmap showing the correlation between KIF20A and tumor-related functional proteins in the TCPA pan-cancer cohort. **(F)** The proteins interacting with KIF20A in different cell sublocations were identified using the ComPPI database. **(G)** Functional enrichment analysis of KIF20A protein in the nucleus. * P < 0.05, ** P < 0.01, *** P < 0.001, **** P < 0.0001.

The previous results showed that the mRNA expression level of KIF20A was up-regulated in tumor tissues. Therefore, we wanted to investigate whether its protein expression level had changed. We obtained the protein expression data of the pan-cancer cohort from the PDC database. The results of differential analysis showed that, compared with adjacent non-cancerous tissues, the protein expression of KIF20A was significantly up-regulated in GBM, HNSC, KIRC, LIHC, LUAD, LUSC, PAAD, and UCEC (**Figure 4A**). Subsequently, we verified this result using the CPPA online tool. The online analysis yielded relatively consistent results. Compared with adjacent non-cancerous tissues, the protein expression of KIF20A was significantly up-regulated in Esophageal Squamous Cell Carcinoma (ESCC), LIHC, STAD, GBM, HNSC, KIRC, LUAD, LUSC, PAAD, and UCEC (**Figure 4B**). In addition, we obtained the immunohistochemical staining scores of tumor tissues from the HPA database. The bar chart showed that the KIF20A protein presented a higher proportion of strong and moderate staining in most tumors. Among them, in cervical cancer, colorectal cancer, ovarian cancer, testicular cancer, and urothelial cancer, the proportion of strong KIF20A staining exceeded 50% (**Figure 4C**). We retrieved the immunohistochemical staining images of these five types of tumor tissues and the normal tissues of the matched homologous organs from the HPA database. The results showed that the staining intensity of KIF20A in tumor tissues was significantly stronger than that in homologous normal tissues, suggesting that the protein expression level of KIF20A is increased in tumors (**Figure 4D**). In addition, in the TCPA pan-cancer cohort, we also investigated the correlation between KIF20A and tumor- related functional proteins. Surprisingly, we found that KIF20A was significantly positively correlated with the cyclin protein CyclinB1 in all tumors except THCA (**Figure 4E**). Finally, we used the ComPPI database to investigate the interacting proteins that have the same subcellular localization as KIF20A (**Figure 4F**). Given the previous results that KIF20A is mainly located in the nucleus, we performed functional enrichment analysis on the interacting proteins located in the nucleus. The results showed that these proteins are jointly involved in a large number of cell cycle-related pathways (**Figure 4G**). The above results indicate that the protein expression of KIF20A is up-regulated in tumor tissues, accelerates the cell cycle and regulates cell division, and may play an important role in tumor proliferation.

### Pathway analysis reveals the regulatory role of KIF20A in tumorigenesis and tumor progression

**Figure 5:**
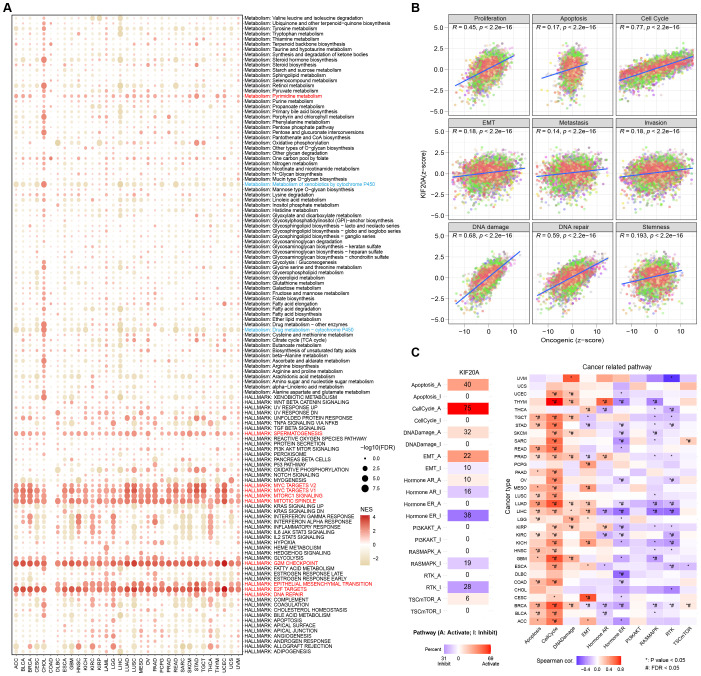
Functional pathway enrichment of KIF20A in tumors and its regulation of key cancer signaling pathways. **(A)** Bubble plots show the GSEA analysis of KIF20A in the TCGA pan-cancer cohort. **(B)** GSVA analysis revealed the relationship between KIF20A expression and tumor-related functional scores. **(C)** Correlation between the expression of KIF20A and the activity of classical oncogenic pathways in GSCA database. * P < 0.05, #FDR<0.05.

In order to clarify the biological functions and molecular mechanisms involved in KIF20A in tumors, we performed pathway enrichment analysis. Firstly, based on the Hallmark gene sets and KEGG metabolism-related gene sets obtained from MSigDB, we carried out GSEA enrichment analysis in the TCGA pan-cancer cohort. Despite the heterogeneity among tumors, relatively consistent results of KIF20A were obtained in some pathways across different types of cancers. For example, we found that DNA repair, E2F targets, epithelial-mesenchymal transition, G2M checkpoint, mitotic spindle, mTORC1 signaling, MYC targets, spermatogenesis, and pyrimidine metabolism were significantly enriched in samples with high KIF20A expression, while metabolism of xenobiotic by cytochrome p450 and drug metabolism-cytochrome p450 were significantly enriched in samples with low KIF20A expression (**Figure 5A**). Subsequently, in order to further understand the mechanism of KIF20A in cancer development, based on the gene sets obtained from the CancerSEA database, we used GSVA analysis to explore the relationship between KIF20A expression and tumor-related functional scores. The results showed that in the TCGA pan-cancer samples, the expression of KIF20A was significantly positively correlated with all tumor-related functions, including proliferation, apoptosis, cell cycle, EMT, metastasis, invasion, DNA damage repair, and stemness (**Figure 5B**). Moreover, KIF20A expression levels showed a significant positive correlation with cell cycle progression across 33 types of cancer (**Supplementary Figure S3A**). Finally, we used the GSCA online tool to explore the regulatory relationship between KIF20A and ten classical oncogenic pathways at the protein level. The results showed that KIF20A is involved in the regulation of oncogenic pathways in most tumors. For example, KIF20A activates the cell cycle pathway in 75% of cancer types and inhibits the hormonal ER pathway in 38% of cancer types (**Figure 5C**; **Supplementary Figure S3B**). The above results indicate that KIF20A is involved in the regulation of various cancer-related functions and pathways and may be a key target for tumor treatment.

### Abnormal epigenetic modifications and genomic mutations are the key mechanisms for the dysregulated expression of KIF20A in tumors

**Figure 6:**
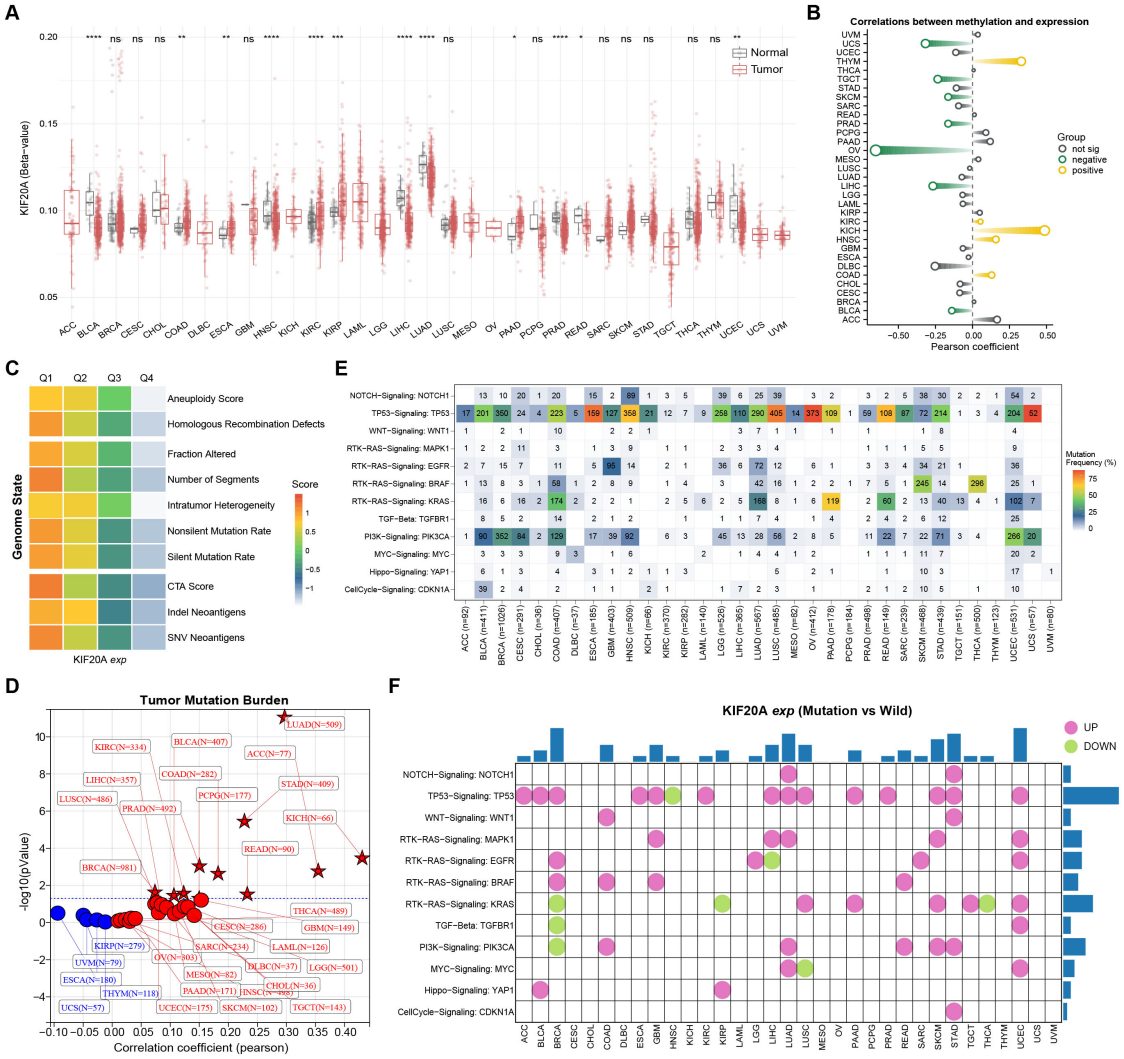
Correlation analysis of KIF20A epigenetic and genomic alterations across pan-cancer types in TCGA. **(A)** Methylation difference analysis of KIF20A in the TCGA pan-cancer cohort. **(B)** Correlation analysis of KIF20A expression and DNA methylation levels across different cancers. **(C)** Relationship between KIF20A and genomic status scores in the TCGA pan-cancer cohort. **(D)** Correlation analysis between KIF20A expression and TMB. **(E)** Heatmap illustrating the mutation frequencies of twelve key molecules in nine classical oncogenic pathways across various cancer types. **(F)** Bubble chart showing the effect of mutations of key molecules on the expression level of KIF20A in different cancer types. * P < 0.05, ** P < 0.01, *** P < 0.001, **** P < 0.0001.

The previous results have confirmed that KIF20A is dysregulated in many tumors and is associated with poor prognosis and clinical progression. To clarify the mechanism of altered KIF20A expression, we delved into the epigenetic and genomic data of the TCGA pan-cancer cohort. Methylation difference analysis showed that, compared with adjacent tissues, KIF20A was hypermethylated in COAD, ESCA, KIRC, KIRP, and PAAD, and hypomethylated in BLCA, HNSC, LIHC, LUAD, PRAD, Rectum Adenocarcinoma (READ), and UCEC (**Figure 6A**). In addition, correlation analysis revealed that the expression of KIF20A was significantly negatively correlated with its methylation level in BLCA, LIHC, Ovarian serous cystadenocarcinoma (OV), PRAD, Skin Cutaneous Melanoma (SKCM), Testicular Germ Cell Tumors (TGCT), and Uterine Carcinosarcoma (UCS), and significantly positively correlated in COAD, HNSC, KICH, KIRC, and Thymoma (THYM) (**Figure 6B**). Subsequently, based on previous studies, we explored the relationship between KIF20A and ten genomic status scores in the TCGA pan-cancer cohort, including aneuploidy score, homologous recombination defects, fraction altered, number of segments, intratumor heterogeneity, nonsilent mutation rate, silent mutation rate, cta score, indel neoantigens and snv neoantigens. We divided the patients into four groups (Q1, Q2, Q3, and Q4) according to the expression of KIF20A, where Q1 represents the 25% of samples with the highest expression level and Q4 represents the 25% of samples with the lowest expression level. The heatmap shows the genomic scores of different grouped samples. We found that as the expression of KIF20A increased, all ten genomic scores gradually increased, suggesting that KIF20A may be closely related to genomic instability (**Figure 6C**). In addition, correlation analysis showed that the expression of KIF20A was significantly positively correlated with TMB in LUAD, ACC, STAD, KICH, READ, COAD, BLCA, Pheochromocytoma and Paraganglioma (PCPG), KIRC, LIHC, LUSC, PRAD, and BRCA (**Figure 6D**). Therefore, we speculate that genomic aberrations in tumors may affect the expression of KIF20A. We incorporated mutation information of nine classical oncogenic pathways in the TCGA pan-cancer genomic data, including NOTCH Signaling, TP53 Signaling, WNT Signaling, RTK-RAS Signaling, TGF-Beta, PI3K Signaling, MYC Signaling, Hippo Signaling and CellCycle Signaling. The heatmap shows the mutation frequencies of twelve key molecules in the nine pathways (**Figure 6E**). Subsequently, we observed the effect of mutations of these key molecules on the expression level of KIF20A. The bubble chart shows that in most tumors, mutations of key molecules mediate changes in the expression of KIF20A. For example, in ACC, BLCA, BRCA, ESCA, GBM, KIRC, LIHC, LUAD, LUSC, PAAD, PRAD, SKCM, STAD, and UCEC, the expression of KIF20A was significantly up-regulated in the TP53 wild-type group compared with the mutant group (**Figure 6F**). The above results indicate that abnormal methylation status and genomic aberrations are important reasons for the dysregulated expression of KIF20A in tumors.

### Multidimensional immunological analyses reveal that KIF20A is a potential target for tumor immunotherapy

**Figure 7:**
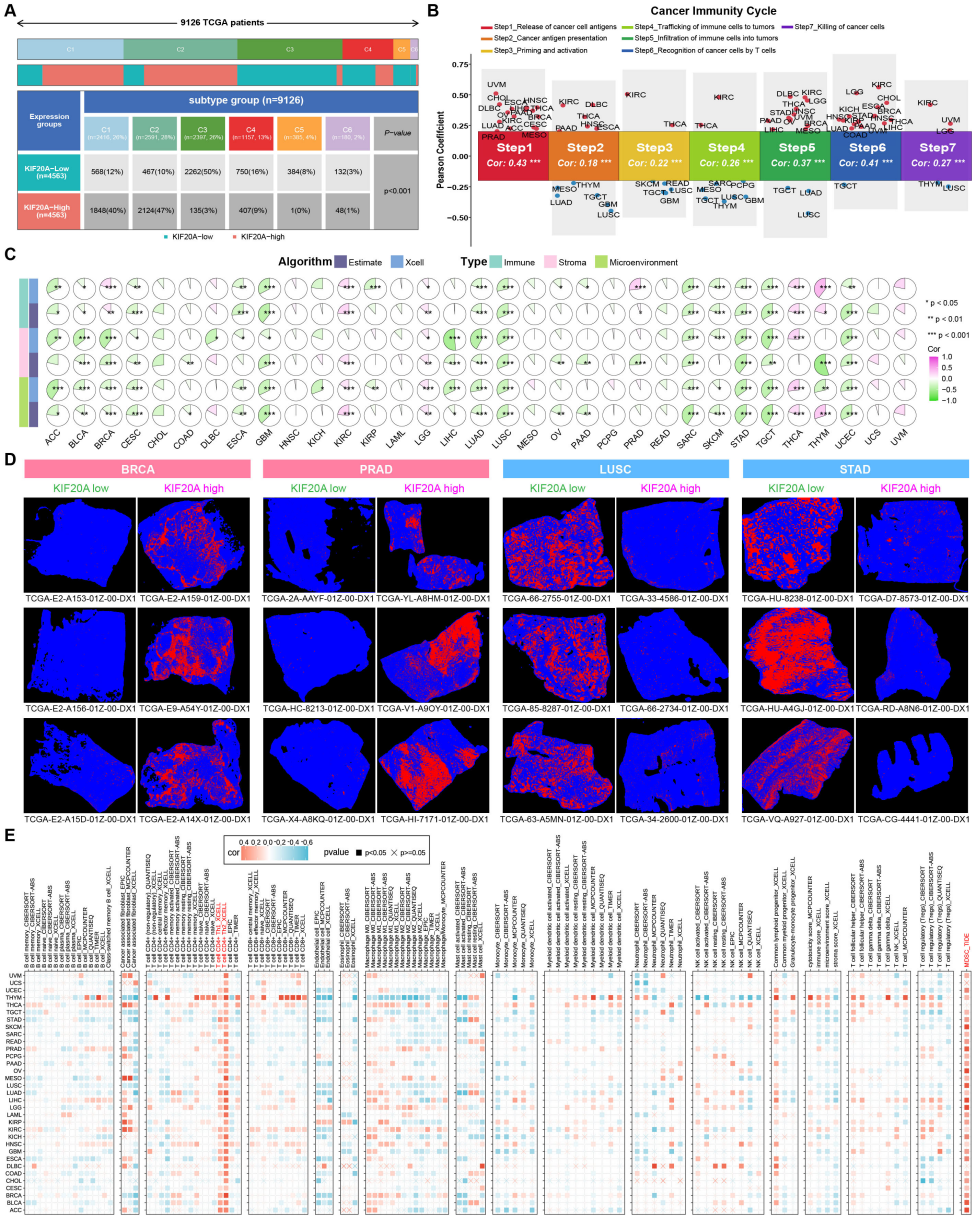
Correlation analysis of KIF20A expression with immune subtypes and the tumor immune microenvironment across all cancer types in TCGA. **(A)** Correlation analysis of KIF20A expression levels with the molecular subtype distribution in TCGA cancer patients. **(B)** Correlation between KIF20A and cancer immune cycle scores. **(C)** Correlation between tumor microenvironment scores and gene expression across different cancer types. **(D)** Tumor lymphocyte infiltration images in the high/low KIF20A expression groups from the TCIA database. **(E)** Correlation analysis between KIF20A expression and the abundance of various immune cells using the TIMER 2.0 database.* P < 0.05, ** P < 0.01, *** P < 0.001.

There is an intricate connection between cancer genomic aberrations and anti-tumor immunity. Given the impact of KIF20A expression on genomic status, we hypothesize its significant role in the tumor immune microenvironment. Therefore, building upon previous studies, we explored the relationship between KIF20A expression and immune subtypes in the TCGA pan-cancer cohort. The results indicated that patients with high KIF20A expression predominantly belonged to subtypes C1 and C2, accounting for 87%; whereas those with low KIF20A expression were mainly in subtypes C3 and C4, representing 66% (**Figure 7A**). Subsequently, using gene sets obtained from the TIP database, we investigated the relationship between KIF20A expression and the anticancer immune cycle, including Step1_Release of cancer cell antigens, Step2_Cancer antigen presentation, Step3_Priming and activation, Step4_Trafficking of immune cells to tumors, Step5_Infiltration of immune cells into tumors, Step6_Recognition of cancer cells by T cells and Step7_Killing of cancer cells. Correlation analysis across pan-cancer samples revealed that KIF20A expression was significantly positively correlated with all seven steps of the scoring (**Supplementary Figure S4A**). Considering the heterogeneity among tumors, we performed correlation analysis between KIF20A expression and the seven-step scores for each type of cancer. The results showed that KIF20A expression was significantly positively correlated with the step1 score in 16 types of cancers such as Uveal melanoma (UVM) and Cholangiocarcinoma (CHOL); significantly positively correlated with the step2 score in 6 types of cancers such as KIRC and Lymphoid Neoplasm Diffuse Large B−cell Lymphoma (DLBC), and significantly negatively correlated with the step2 score in 6 types of cancers such as LUSC and GBM; significantly positively correlated with the step3 score in KIRC and Thyroid carcinoma (THCA), and significantly negatively correlated with the step3 score in 5 types of cancers such as TGCT and GBM; significantly positively correlated with the step4 score in KIRC and THCA, and significantly negatively correlated with the step4 score in 7 types of cancers such as THYM and GBM; significantly positively correlated with the step5 score in 12 types of cancers such as KIRC and DLBC, and significantly negatively correlated with the step5 score in 3 types of cancers such as LUSC and LUAD; significantly positively correlated with the step6 score in 16 types of cancers such as KIRC and Brain Lower Grade Glioma (LGG), and significantly negatively correlated with the step6 score in TGCT; significantly positively correlated with the step7 score in KIRC, UVM, and LGG, and significantly negatively correlated with the step7 score in LUSC and THYM (**Figure 7B**). Additionally, we evaluated the correlation between KIF20A expression and tumor immune microenvironment scores, including immune scores, stromal scores, and microenvironment scores, using the Estimate and Xcell algorithms in the pan-cancer cohort. The results from both algorithms were highly consistent, demonstrating that KIF20A expression was significantly negatively correlated with immune and stromal scores in most tumors (**Figure 7C**). To validate the microenvironmental analysis, we obtained tumor lymphocyte infiltration images from the TCIA database for TCGA samples. We selected four representative cancer types, including BRCA, PRAD, LUSC, and STAD. The results were consistent with the microenvironmental analysis: in BRCA and PRAD, samples with high KIF20A expression had more extensive immune cell infiltration; in LUSC and STAD, samples with low KIF20A expression had more extensive immune cell infiltration (**Figure 7D**). Finally, we used the TIMER 2.0 database to assess the correlation between KIF20A and immune cells. The results showed that in the pan-cancer cohort, KIF20A expression was negatively correlated with the abundance of the vast majority of immune cells. Notably, we observed that KIF20A exhibited significant positive correlations with Th1 cells, Th2 cells, and myeloid-derived suppressor cells (MDSCs) in nearly all tumor types, suggesting that KIF20A plays a role in shaping the immune microenvironment (**Figure 7E**). The aforementioned results indicate that KIF20A expression affects the immune status of patients and may play a significant role in antitumor immune responses.

**Figure 8:**
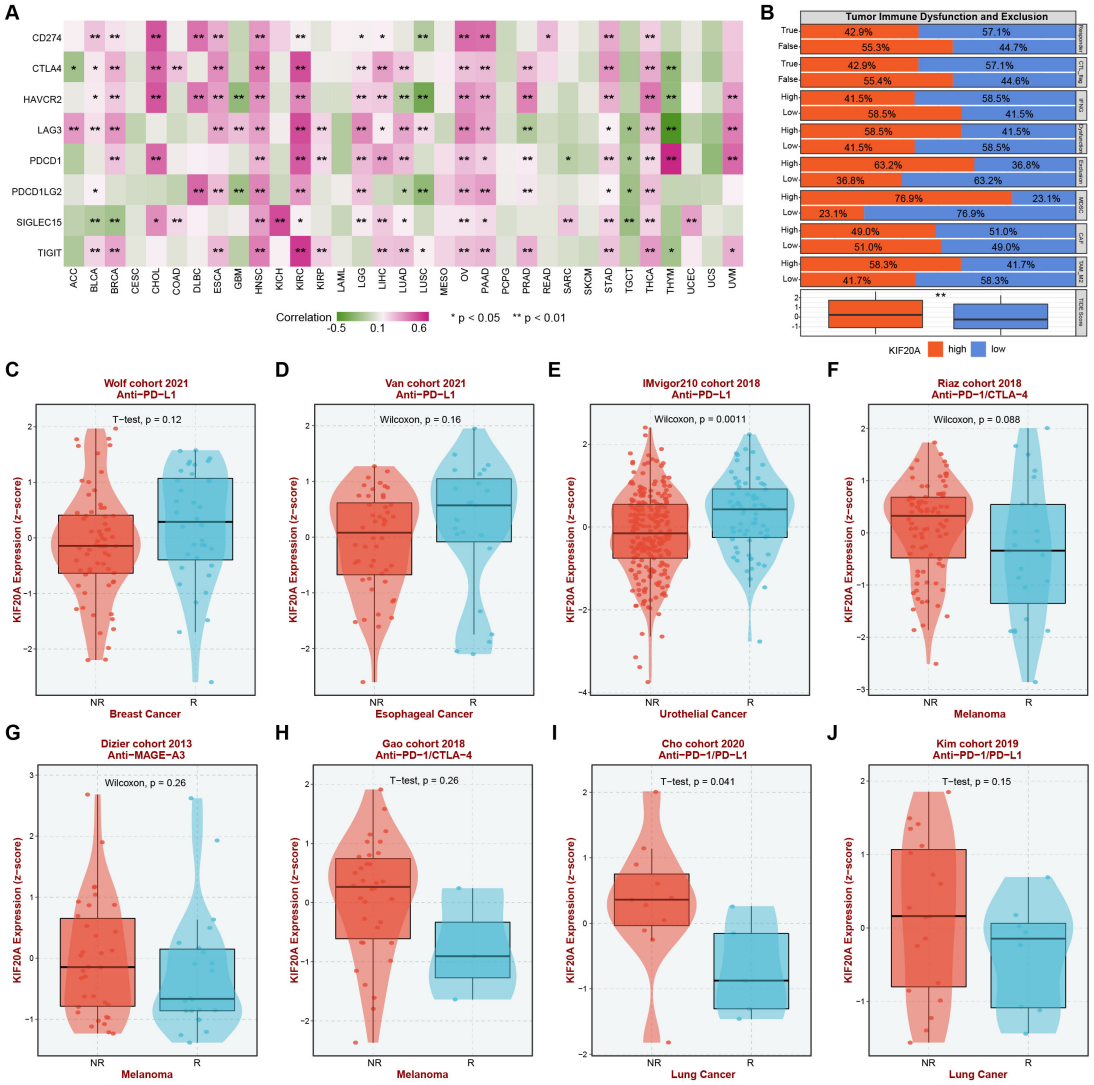
Correlation analysis of KIF20A with immune checkpoint expression and immune therapy response. **(A)** Heatmap showing the correlation between KIF20A and eight common clinical immune checkpoints in TCGA pan-cancer samples. **(B)** Correlation between KIF20A expression and immunotherapy markers in the TIDE databa. **(C-J)** Differential response to immune checkpoint inhibitor treatment based on KIF20A gene expression across different cancer cohorts. * P < 0.05, ** P < 0.01.

The status of the immune microenvironment and the expression of immune checkpoints play a crucial role in the response to immunotherapy. Therefore, we explored the correlation between the expression of KIF20A and eight commonly used clinical immune checkpoints in the TCGA pan-cancer cohort, including CD274, CTLA4, HAVCR2, LAG3, PDCD1, PDCD1LG2, SIGLEC15 AND TIGIT. The results showed that KIF20A was significantly positively correlated with the expression of immune checkpoints in most tumors. For example, in HNSC, KIRC, OV, PAAD, STAD and THCA, the expression of KIF20A was significantly positively correlated with all eight immune checkpoints. However, in GBM, LUSC, TGCT, and THYM, KIF20A expression was negatively correlated with the expression of several immune checkpoints (**Figure 8A**). Subsequently, based on the scores of immune markers obtained from the TIDE database, we predicted the impact of KIF20A on immunotherapy in pan-cancer samples. The results demonstrated that in the non-responder group to treatment, CTL-negative group, low IFNG score group, high dysregulation and exclusion score group, as well as the group with high abundances of MDSC and TAM_M2 cells, there was a higher proportion of patients with high expression of KIF20A. Overall, we found that patients with high expression of KIF20A had higher TIDE scores, suggesting that high expression of KIF20A might be associated with resistance to immunotherapy (**Figure 8B**). Considering the heterogeneity among tumors and the limitations of the prediction results, we evaluated the impact of KIF20A on immunotherapy in real-world immunotherapy cohorts. It is worth noting that the relationship between KIF20A expression and immune therapy response shows heterogeneity across different cancer types. For example, in breast cancer and esophageal cancer, low expression of KIF20A is associated with poor treatment response, whereas in melanoma and lung cancer, high expression of KIF20A is linked to treatment resistance. This discrepancy may arise from differences in the immune microenvironment composition, the background of driving mutations, or variations in treatment regimens, suggesting that the predictive value of KIF20A may be tumor type-specific (**Figure 8C-J**), with ROC curve diagnostic areas exceeding 0.6 in all cases (**Supplementary Figure S4B**). The above results suggest that KIF20A plays a role in the regulation of immune checkpoint expression in most tumors and is associated with immunotherapy efficacy. However, this effect demonstrates heterogeneity across different cancer types.

### Association analysis of KIF20A with the tumour immune microenvironment

**Figure 9:**
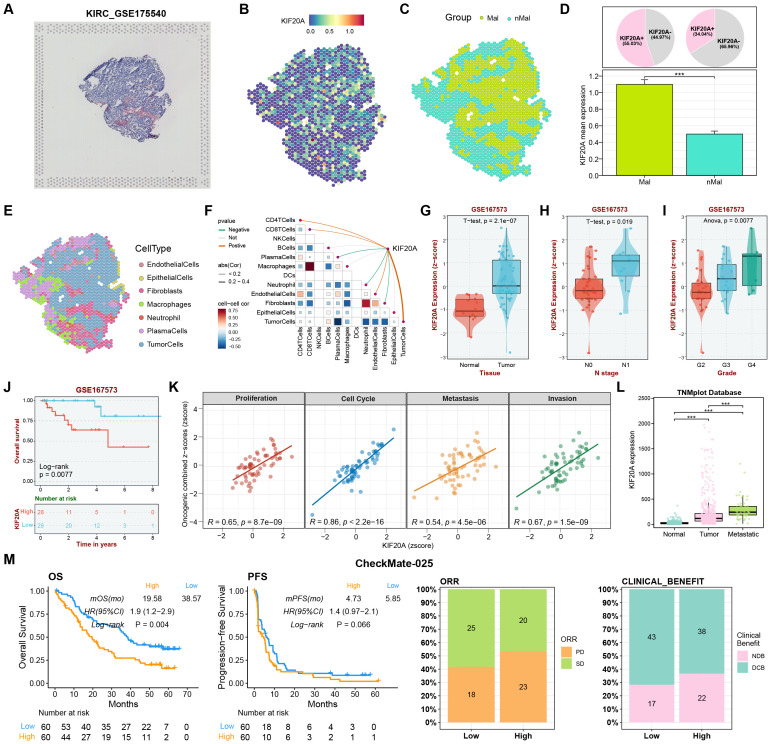
Comprehensive analysis of KIF20A in KIRC highlighting its expression, correlation with clinical parameters, and impact on immunotherapy response. **(A)**HE staining of KIRC tissue section from the SpatialTME database. **(B)** Spatial expression pattern of KIF20A in KIRC tissue. **(C)** Malignant and non-malignant areas in KIRC tissue. **(D)** Expression of KIF20A in malignant and non-malignant regions. **(E)** Cell type deconvolution of KIRC tissue at spatial transcriptome resolution. **(F)** Correlation between KIF20A expression and microenvironment cell content at spatial transcriptome resolution. **(G)** The expression difference of KIF20A between normal tissues and tumor tissues in GSE167573 dataset. **(H)** Relationship between KIF20A expression and lymph node metastasis in GSE167573 dataset. **(I)** Relationship between KIF20A expression and histological grade in GSE167573 dataset. **(J)** Survival analysis based on KIF20A expression level in the GSE167573 dataset. **(K)** Association of KIF20A with OS, PFS, ORR, and clinical response rate in the CheckMate025 cohort.

Based on the previous research results, we found that KIF20A underwent multi-omics alterations including upregulated expression and copy number amplification in KIRC cells and tissues, and its high expression was closely associated with advanced staging, poor tissue differentiation and poor prognosis. Therefore, we verified the function and mechanism of KIF20A using additional KIRC cohorts and *in vitro* and *in vivo* experiments. Firstly, we explored the spatial transcriptome data of KIRC using the SpatialTME database. Based on high-definition HE-stained KIRC tissue sections, we mapped the spatial expression information of KIF20A (**Figures 9A, B**). We found that the expression of KIF20A in malignant regions was significantly higher than that in non-malignant regions, and the proportion of KIF20A-positive spots in malignant regions was also higher than that in non-malignant regions (**Figure 9C, D**). In addition, after deconvoluting the spatial spots into specific cell types, correlation analysis showed that the expression of KIF20A was significantly positively correlated with the contents of CD4 T cells, CD8 T cells, endothelial cells and tumor cells, suggesting that the highly expressed KIF20A in tumor cells might be involved in the regulation of the immune microenvironment (**Figures 9E, F**). In the KIRC datasets from the GEO database, we found that: the expression of KIF20A in tumor tissues was significantly higher than that in normal tissues; compared with lymph node-negative samples, the expression of KIF20A was upregulated in lymph node metastasis samples; as the degree of differentiation decreased, the expression of KIF20A gradually increased; patients with high expression of KIF20A had a shorter OS; the expression of KIF20A was significantly positively correlated with proliferation, cell cycle, metastasis and invasion (**Figures 9J, K**). Subsequently, we verified the relationship between KIF20A and the progression of renal cancer in the TNMplot database. The results showed that the expression of KIF20A was gradually upregulated during the process from normal to tumor and then to metastasis (**Figure 9L**). Finally, we explored the role of KIF20A in the response to immunotherapy for advanced renal cancer in the CheckMate 025 study. The results showed that patients with high expression of KIF20A had a shorter survival period, including OS and PFS. The analysis of objective response rate showed that there were more patients with disease progression in the group with high expression of KIF20A. The analysis of clinical benefit rate also showed that there were more patients with no durable benefit (NDB) in the group with high expression of KIF20A (**Figure 9M**). The above results clarify the oncogenic role of KIF20A in KIRC and suggest that it is a potential target for immunotherapy.

### *In vitro* and *in vivo* experimental validation

**Figure 10:**
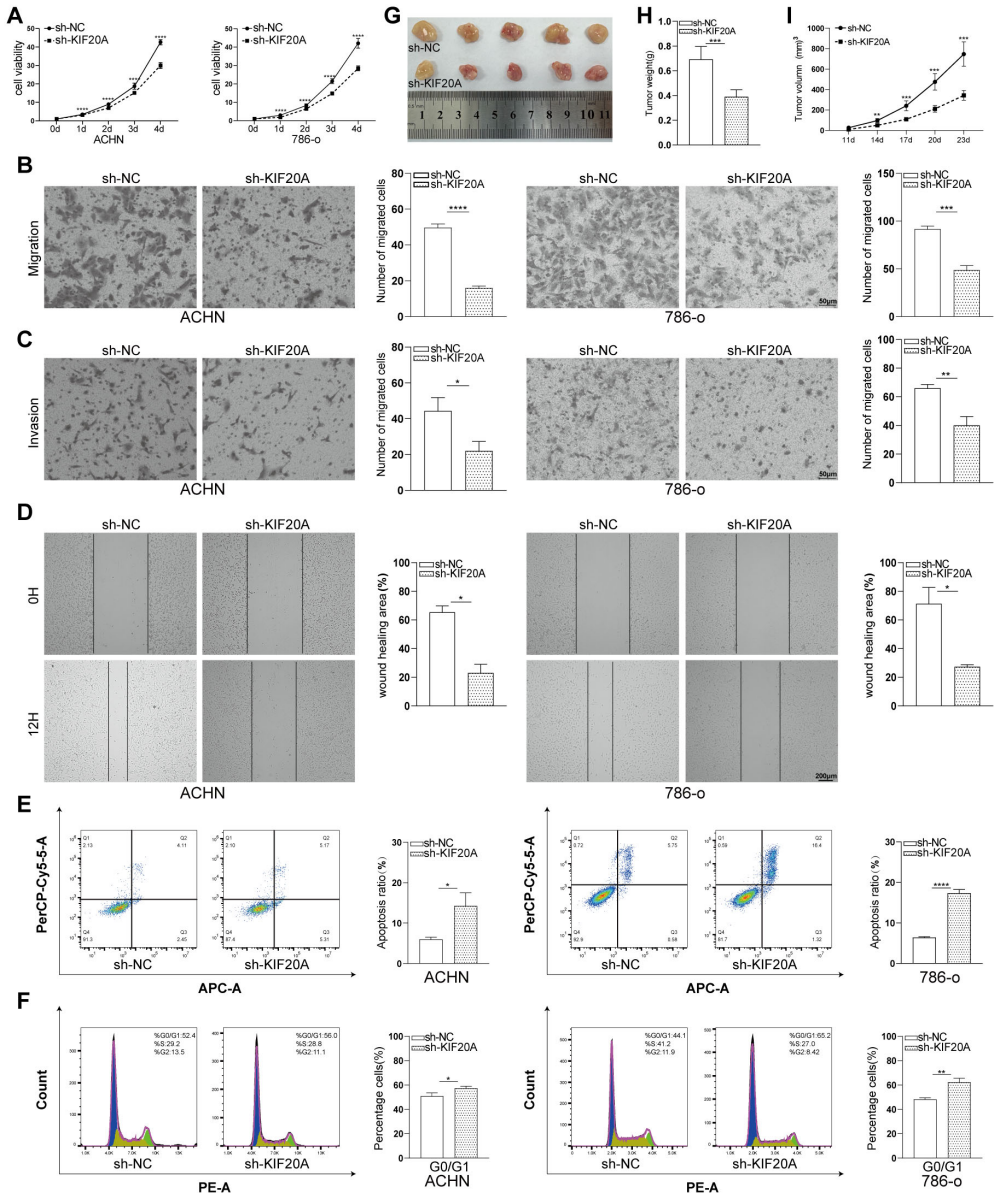
Downregulating KIF20A inhibits the proliferation and metastasis of ccRCC. **(A)** Analysis of cell survival curves under different treatment methods. **(B, C)** Using the Transwell assay to investigate the effects of KIF20A knockdown on cell migration and invasion. **(D)** Wound healing assay to evaluate the effect of KIF20A knockdown on cell migration, with wound closure rates measured after 12 hours of incubation. **(E, F)** Flow cytometry analysis of apoptosis and cell cycle progression in sh-NC and sh-KIF20A groups. **(G-I)** ACHN cells from the control group and KIF20A knockdown group were subcutaneously injected into nude mice. Tumor volume, growth status, and weight were measured at designated time points (^*^P < 0.05, ^**^P < 0.01, ^***^P < 0.001, ^****^P < 0.0001).

Based on the above bioinformatics analysis, KIF20A was found to influence tumor growth, metastasis, invasion, and cell cycle progression across multiple cancer types. We then used renal clear cell carcinoma as a model to further validate these findings through *in vivo* and *in vitro* experiments. We knocked down KIF20A in two renal clear cell carcinoma cell lines, 786-o and ACHN (**Supplementary Figure S5A, B**), to determine whether it affects the malignant biological behavior of tumor cells. CCK8 cell proliferation assay results demonstrated that KIF20A reduction significantly inhibited proliferation in both 786-o and ACHN cells (**Figure 10A**). Subsequently, Transwell and wound-healing assays were conducted to evaluate migration and invasion capabilities in 786-o and ACHN cells following KIF20A knockdown. Results revealed that tumor cell migration and invasion capabilities were significantly reduced following KIF20A gene knockdown (**Figures 10B-D**). Additionally, flow cytometry was employed to examine the effects of KIF20A knockdown on apoptosis and cell cycle progression. Apoptosis assays demonstrated a markedly increased proportion of apoptotic cells in both cell lines compared to the control group (**Figure 10E**). Cell cycle analysis revealed a significant increase in the proportion of 786-o and ACHN cells arrested in the G1 phase in the KIF20A-knockdown group compared to the control group. This indicates that inhibiting KIF20A can suppress tumor cell progression by arresting cells in the G1 phase, thereby inhibiting cell proliferation (**Figure 10F**). Finally, xenograft tumor experiments in nude mice confirmed that compared to the control group, the KIF20A knockdown group exhibited significantly reduced tumor volume, growth rate, and weight in nude mice (**Figure 10G-I**).

## Discussion

Cancer remains a major threat to human health, with persistently low five-year survival rates for most malignancies despite advances in detection and treatment (27, 28). This underscores the need for innovative diagnostic and therapeutic strategies. Pan-cancer analysis provides a powerful tool for identifying shared and distinct molecular features across cancer types, offering new insights for drug development, prevention, and personalized treatment. The TCGA database, containing multi-omics data from 33 tumor types, is a valuable resource for exploring genomic alterations in cancer (29). To investigate the role of KIF20A in cancer, we performed a pan-cancer analysis to evaluate its diagnostic and prognostic potential, as well as its involvement in tumor initiation and progression.

In this study, we conducted the first comprehensive pan-cancer multi-omics analysis of KIF20A, revealing its critical role in tumorigenesis, immune regulation, and clinical prognosis across diverse cancer types. Our findings demonstrate that KIF20A is frequently overexpressed at both mRNA and protein levels in multiple malignancies, driven by epigenetic alterations and genomic instability, and is strongly associated with advanced disease stage, poor differentiation, and unfavorable patient outcomes.

KIF20A, a nucleus-localized kinesin, plays a central role in cell cycle progression, particularly during the G2/M phase (16, 30). This aligns with its known function in cytokinesis and spindle organization, and our functional enrichment analyses further confirmed its involvement in cell cycle-related pathways, DNA repair, and mitotic processes. Our findings confirm and broadly validate the proliferative and metastatic roles of KIF20A previously observed in individual cancer studies, including hepatocellular carcinoma (HCC), glioma, and colorectal cancer. More importantly, our pan-cancer analysis reveals that these functions are not isolated events but are driven by conserved mechanisms that operate across a wide range of cancer types.

Notably, we uncovered a significant association between KIF20A expression and the tumor immune microenvironment. KIF20A expression levels are associated with immune characteristics, particularly with increased infiltration of Th2 cells and MDSCs in nearly all cancer types. Additionally, KIF20A is correlated with the expression levels of several immune checkpoint molecules, including PD-L1, CTLA-4, and LAG-3, across most tumor types. Based on the single-cell results, KIF20A is predominantly highly expressed in malignant cells across various cancers, with very low expression in immune cells. Additionally, the spatial transcriptomics data from KIRC reveal that KIF20A is highly expressed in the tumor regions and shows a significant correlation with immune cells. We hypothesize that the immune enrichment is due to spatial proximity rather than the expression of KIF20A in immune cells.

However, this study also reveals a significant heterogeneity in the relationship between KIF20A and immune activation. In certain cancer types, such as KIRC and THCA, high expression of KIF20A is positively correlated with immune cell infiltration and immune activation. Conversely, in other types, such as LUSC and GBM, this correlation is negative. This disparity may arise from multiple factors. Firstly, the immune microenvironment across different cancer types is inherently distinct, encompassing variations in immune cell composition, cytokine profiles, and immune suppression mechanisms (31, 32). For instance, in “hot” tumors where immune activity is robust, KIF20A may enhance immune recognition indirectly by promoting tumor cell proliferation and antigen release. In contrast, in “cold” tumors or those with significant immune suppression, KIF20A may predominantly drive the tumor’s intrinsic immune evasion mechanisms. Secondly, KIF20A might exert differential effects on immune cell functions in various environments by modulating distinct downstream signaling pathways such as JAK-STAT, PI3K-AKT, or cell cycle pathways. Additionally, variations in tumor mutational burden, microsatellite instability status, and epigenetic modifications may collectively shape the context of KIF20A’s interaction with the immune system. Future studies should leverage single-cell transcriptomics, spatial proteomics, and *in vitro* immune co-culture models to further elucidate the specific mechanisms by which KIF20A operates within different immune microenvironments.

Through *in vitro* experiments, we validated that KIF20A knockdown inhibits the proliferation, migration, and invasion capabilities of renal cell carcinoma cells, further confirming its pivotal role in tumor invasiveness. Concurrently, results from nude mouse experiments demonstrated that KIF20A knockdown suppresses tumor growth rates in nude mice. Combined with bioinformatics evidence from spatial transcriptomics and immunotherapy cohorts, these findings strongly support KIF20A as a promising therapeutic target.

Despite the significant insights gained, this study has several limitations. It primarily relies on retrospective omics data from public databases, which may not fully capture the intratumoral heterogeneity and dynamic changes before and after treatment. Therefore, our conclusions need to be validated in prospective clinical cohorts. Although we have predicted the role of KIF20A in immunotherapy through various algorithms, these computational predictions still require functional validation using *in vivo* and *in vitro* immunotherapy models, such as humanized mouse models and immune cell co-culture systems. While this study highlights the pan-cancer relevance of KIF20A, the specific molecular mechanisms may vary across different cancer types. Current research has not fully elucidated how KIF20A regulates specific signaling pathways or intercellular communication in different immune contexts. The accessibility of KIF20A as a therapeutic target, the design of specific inhibitors, and its combinatorial strategies with existing immunotherapies warrant further investigation in future studies.

In summary, our multi-omics analysis identifies KIF20A as a key oncogene with dual roles in promoting cell cycle progression and modulating the immune microenvironment. These findings provide strong evidence supporting the targeting of KIF20A in combination immunotherapies, thereby opening new avenues for precision oncology.

## Data Availability

The datasets presented in this study can be found in online repositories. The names of the repository/repositories and accession number(s) can be found in the article/Supplementary Material.

## References

[1] Bray F Laversanne M Sung H Ferlay J Siegel RL Soerjomataram I . Global cancer statistics 2022: GLOBOCAN estimates of incidence and mortality worldwide for 36 cancers in 185 countries. CA: Cancer J Clin. (2024) 74:229–63. doi: 10.3322/caac.21834, PMID: 38572751

[2] Lone SN Nisar S Masoodi T Singh M Rizwan A Hashem S . Liquid biopsy: a step closer to transform diagnosis, prognosis and future of cancer treatments. Mol Cancer. (2022) 21:79. doi: 10.1186/s12943-022-01543-7, PMID: 35303879 PMC8932066

[3] Yang M Zhao Y Li C Weng X Li Z Guo W . Multimodal integration of liquid biopsy and radiology for the noninvasive diagnosis of gallbladder cancer and benign disorders. Cancer Cell. (2025) 43:398–412.e4. doi: 10.1016/j.ccell.2025.02.011, PMID: 40068597

[4] Motohara T Masuda K Morotti M Zheng Y El-Sahhar S Chong KY . An evolving story of the metastatic voyage of ovarian cancer cells: cellular and molecular orchestration of the adipose-rich metastatic microenvironment. Oncogene. (2019) 38:2885–98. doi: 10.1038/s41388-018-0637-x, PMID: 30568223 PMC6755962

[5] Cui E Guo H Shen M Yu H Gu D Mao W . Adiponectin inhibits migration and invasion by reversing epithelial−mesenchymal transition in non−small cell lung carcinoma. Oncol Rep. (2018) 40:1330–8. doi: 10.3892/or.2018.6523, PMID: 29956809 PMC6072398

[6] Panizza BJ de Souza P Cooper A Roohullah A Karapetis CS Lickliter JD . Phase I dose-escalation study to determine the safety, tolerability, preliminary efficacy and pharmacokinetics of an intratumoral injection of tigilanol tiglate (EBC-46). EBioMedicine. (2019) 50:433–41. doi: 10.1016/j.ebiom.2019.11.037, PMID: 31810818 PMC6921293

[7] Liu H Tang L Li Y Xie W Zhang L Tang H . Nasopharyngeal carcinoma: current views on the tumor microenvironment's impact on drug resistance and clinical outcomes. Mol Cancer. (2024) 23:20. doi: 10.1186/s12943-023-01928-2, PMID: 38254110 PMC10802008

[8] Xu X Song Y Li M Liu F Zhang H Xu J . Biomimetic tumor cell membrane-camouflaged nanomicelles for synergistic chemo-immunotherapy of Triple-negative breast cancer. Mater Today Bio. (2025) 33:102012. doi: 10.1016/j.mtbio.2025.102012, PMID: 40677402 PMC12268940

[9] Yan Z Zhang Z Chen Y Xu J Wang J Wang Z . Enhancing cancer therapy: the integration of oncolytic virus therapy with diverse treatments. Cancer Cell Int. (2024) 24:242. doi: 10.1186/s12935-024-03424-z, PMID: 38992667 PMC11238399

[10] Mayer-Suess L Knoflach M Pircher A Kiechl S Schmidauer C Hametner E . Case report: Dissolving carotid plaque associated to Lorlatinib-related dyslipidemia. Front Oncol. (2024) 14:1322501. doi: 10.3389/fonc.2024.1322501, PMID: 38505589 PMC10949858

[11] Zhang S Lv K Liu Z Zhao R Li F . Fatty acid metabolism of immune cells: a new target of tumour immunotherapy. Cell Death Discov. (2024) 10:39. doi: 10.1038/s41420-024-01807-9, PMID: 38245525 PMC10799907

[12] Wang D Wang T Yu H Feng B Zhou L Zhou F . Engineering nanoparticles to locally activate T cells in the tumor microenvironment. Sci Immunol. (2019) 4(37):eaau6584. doi: 10.1126/sciimmunol.aau6584, PMID: 31300478

[13] Qin Y Liu Y Xiang X Long X Chen Z Huang X . Cuproptosis correlates with immunosuppressive tumor microenvironment based on pan-cancer multiomics and single-cell sequencing analysis. Mol Cancer. (2023) 22:59. doi: 10.1186/s12943-023-01752-8, PMID: 36959665 PMC10037895

[14] Zhou X Yao G Zhang J Bian J Li G Xu J . An integrated multi-omics analysis of topoisomerase family in pan-cancer: Friend or foe? PloS One. (2022) 17:e0274546. doi: 10.1371/journal.pone.0274546, PMID: 36288358 PMC9604985

[15] Zhao X Zhou LL Li X Ni J Chen P Ma R . Overexpression of KIF20A confers Malignant phenotype of lung adenocarcinoma by promoting cell proliferation and inhibiting apoptosis. Cancer Med. (2018) 7:4678–89. doi: 10.1002/cam4.1710, PMID: 30105795 PMC6143951

[16] Wu WD Yu KW Zhong N Xiao Y She ZY . Roles and mechanisms of Kinesin-6 KIF20A in spindle organization during cell division. Eur J Cell Biol. (2019) 98:74–80. doi: 10.1016/j.ejcb.2018.12.002, PMID: 30579662

[17] Jin Z Peng F Zhang C Tao S Xu D Zhu Z . Expression, regulating mechanism and therapeutic target of KIF20A in multiple cancer. Heliyon. (2023) 9:e13195. doi: 10.1016/j.heliyon.2023.e13195, PMID: 36798768 PMC9925975

[18] Wang Z Shi D Li X Wang X Bai J . KIF20A promotes triple-negative breast cancer progression via activation of the IL-17 signaling pathway. Biochem Biophys Res Commun. (2025) 770:152031. doi: 10.1016/j.bbrc.2025.152031, PMID: 40393105

[19] Wang Q Wu H Wu Q Zhong S . Berberine targets KIF20A and CCNE2 to inhibit the progression of nonsmall cell lung cancer via the PI3K/AKT pathway. Drug Dev Res. (2023) 84:907–21. doi: 10.1002/ddr.22061, PMID: 37070571

[20] Chen S Zhao L Liu J Han P Jiang W Liu Y . Inhibition of KIF20A enhances the immunotherapeutic effect of hepatocellular carcinoma by enhancing c-Myc ubiquitination. Cancer Lett. (2024) 598:217105. doi: 10.1016/j.canlet.2024.217105, PMID: 38971490

[21] Meng X Li W Yuan H Dong W Xiao W Zhang X . KDELR2-KIF20A axis facilitates bladder cancer growth and metastasis by enhancing Golgi-mediated secretion. Biol Procedures. (2022) 24:12. doi: 10.1186/s12575-022-00174-y, PMID: 36096734 PMC9465899

[22] Xiong M Zhuang K Luo Y Lai Q Luo X Fang Y . KIF20A promotes cellular Malignant behavior and enhances resistance to chemotherapy in colorectal cancer through regulation of the JAK/STAT3 signaling pathway. Aging. (2019) 11:11905–21. doi: 10.18632/aging.102505, PMID: 31841120 PMC6949076

[23] Tang J Xu J Zhi Z Wang X Wang Y Zhou Y . MiR-876-3p targets KIF20A to block JAK2/STAT3 pathway in glioma. Am J Trans Res. (2019) 11:4957–66., PMID: 31497212 PMC6731397

[24] Kawai Y Shibata K Sakata J Suzuki S Utsumi F Niimi K . KIF20A expression as a prognostic indicator and its possible involvement in the proliferation of ovarian clear−cell carcinoma cells. Oncol Rep. (2018) 40:195–205. doi: 10.3892/or.2018.6401, PMID: 29749467 PMC6059742

[25] Lee JS Ruppin E . Multiomics prediction of response rates to therapies to inhibit programmed cell death 1 and programmed cell death 1 ligand 1. JAMA Oncol. (2019) 5:1614–8. doi: 10.1001/jamaoncol.2019.2311, PMID: 31436822 PMC6707018

[26] Thorsson V Gibbs DL Brown SD Wolf D Bortone DS Ou Yang TH . The immune landscape of cancer. Immunity. (2018) 48:812–30.e14. doi: 10.1016/j.immuni.2018.03.023, PMID: 29628290 PMC5982584

[27] Chen GM Chen C Das RK Gao P Chen CH Bandyopadhyay S . Integrative bulk and single-cell profiling of premanufacture T-cell populations reveals factors mediating long-term persistence of CAR T-cell therapy. Cancer Discov. (2021) 11:2186–99. doi: 10.1158/2159-8290.CD-20-1677, PMID: 33820778 PMC8419030

[28] Nahshon C Barnett-Griness O Segev Y Schmidt M Ostrovsky L Lavie O . Five-year survival decreases over time in patients with BRCA-mutated ovarian cancer: a systemic review and meta-analysis. Int J Gynecol Cancer: Off J Int Gynecol Cancer Society. (2022) 32:48–54. doi: 10.1136/ijgc-2020-001392, PMID: 32522775

[29] Huang C He J Dong Y Huang L Chen Y Peng A . Identification of novel prognostic markers associated with laryngeal squamous cell carcinoma using comprehensive analysis. Front Oncol. (2021) 11:779153. doi: 10.3389/fonc.2021.779153, PMID: 35087752 PMC8787159

[30] Wei J Chen X Li Y Li R Bao K Liao L . Cucurbitacin B-induced G2/M cell cycle arrest of conjunctival melanoma cells mediated by GRP78-FOXM1-KIF20A pathway. Acta Pharm Sin B. (2022) 12:3861–76. doi: 10.1016/j.apsb.2022.05.021, PMID: 36213538 PMC9532536

[31] Massagué J Ganesh K . Metastasis-initiating cells and ecosystems. Cancer Discov. (2021) 11:971–94. doi: 10.1158/2159-8290.CD-21-0010, PMID: 33811127 PMC8030695

[32] Montagne JM Jaffee EM Fertig EJ . Multiomics empowers predictive pancreatic cancer immunotherapy. J Immunol (Baltimore Md: 1950). (2023) 210:859–68. doi: 10.4049/jimmunol.2200660, PMID: 36947820 PMC10236355

