# Supplementary figures and images for: Targeting KIF20A: a new frontier in cancer treatment revealed by multi-omics analysis
Source: Front Immunol. 2026 Jan 21;17:1744899. doi: 10.3389/fimmu.2026.1744899 (PMC12867832; doi:10.3389/fimmu.2026.1744899)

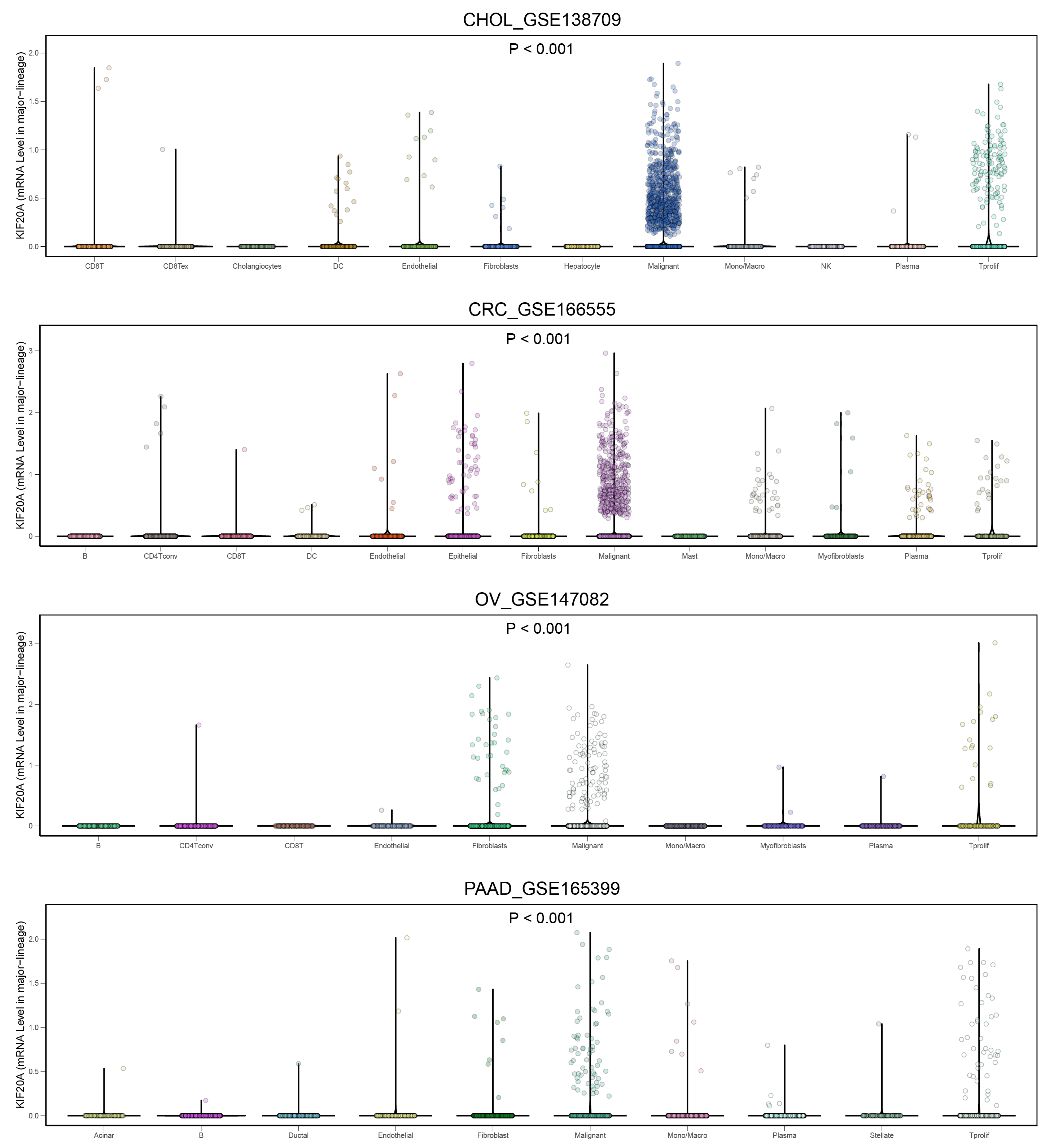

Supplement: Supplementary file 2 [file Image1.tif]

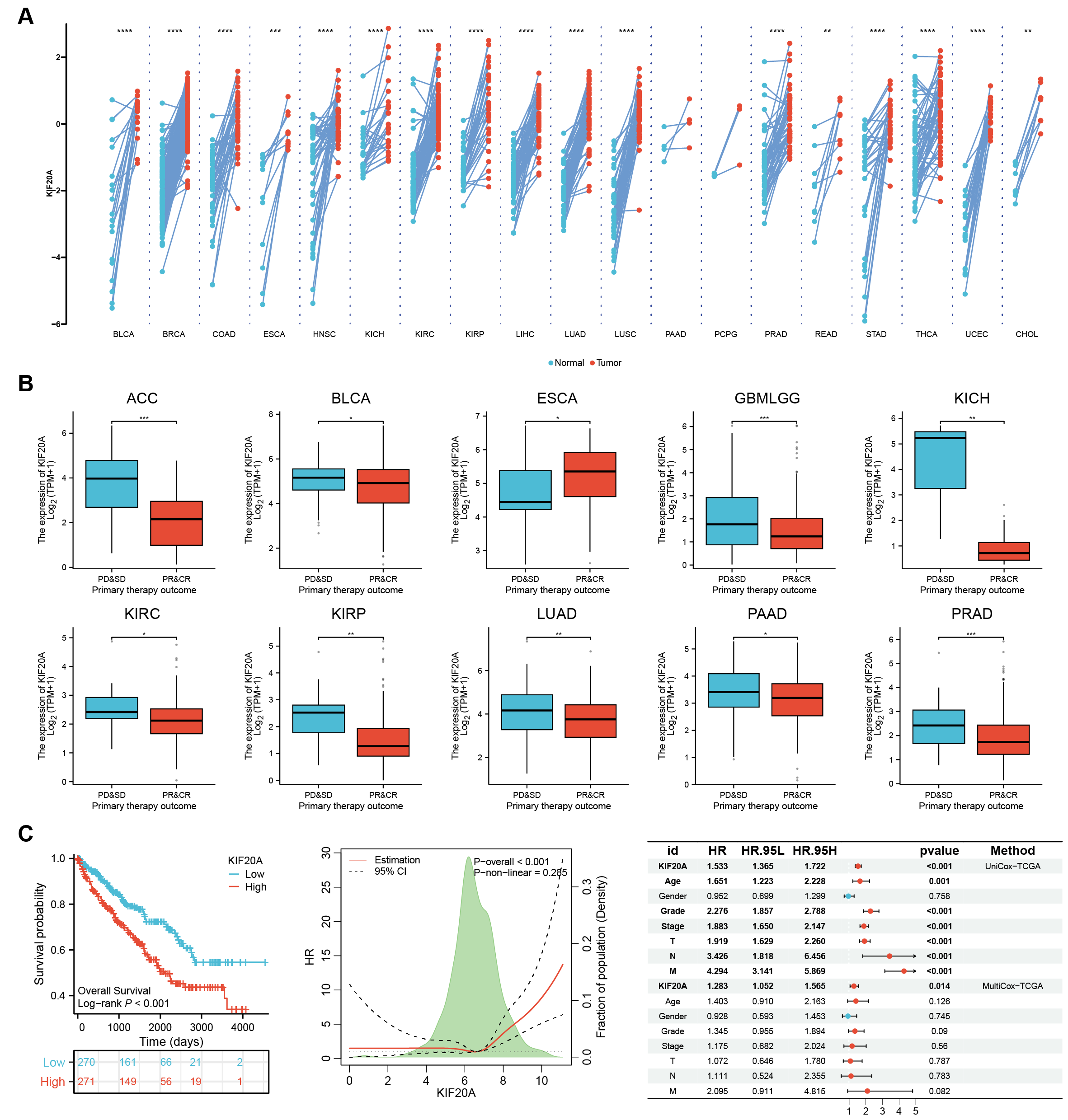

Supplement: Supplementary file 3 [file Image2.tif]

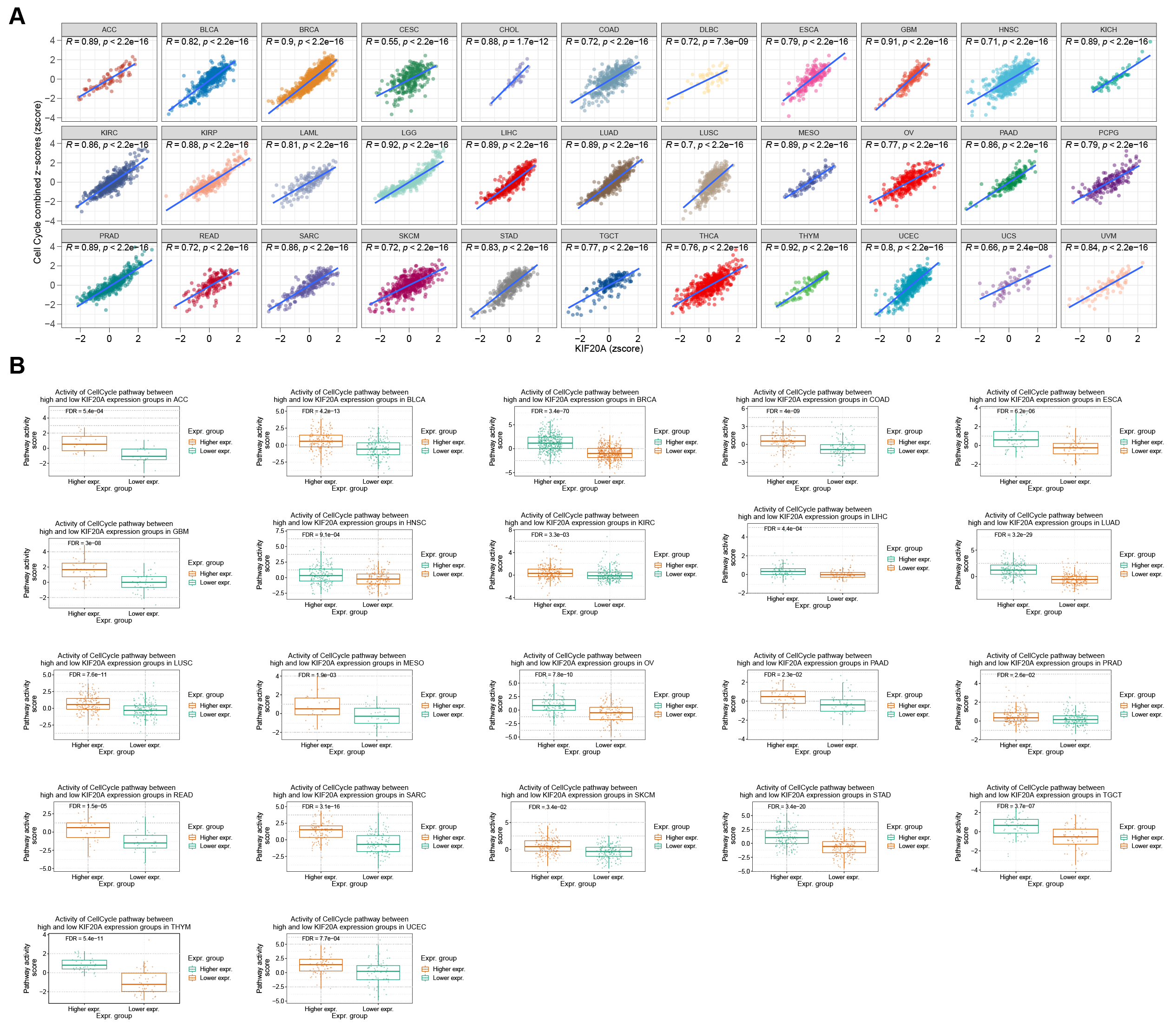

Supplement: Supplementary file 4 [file Image3.tif]

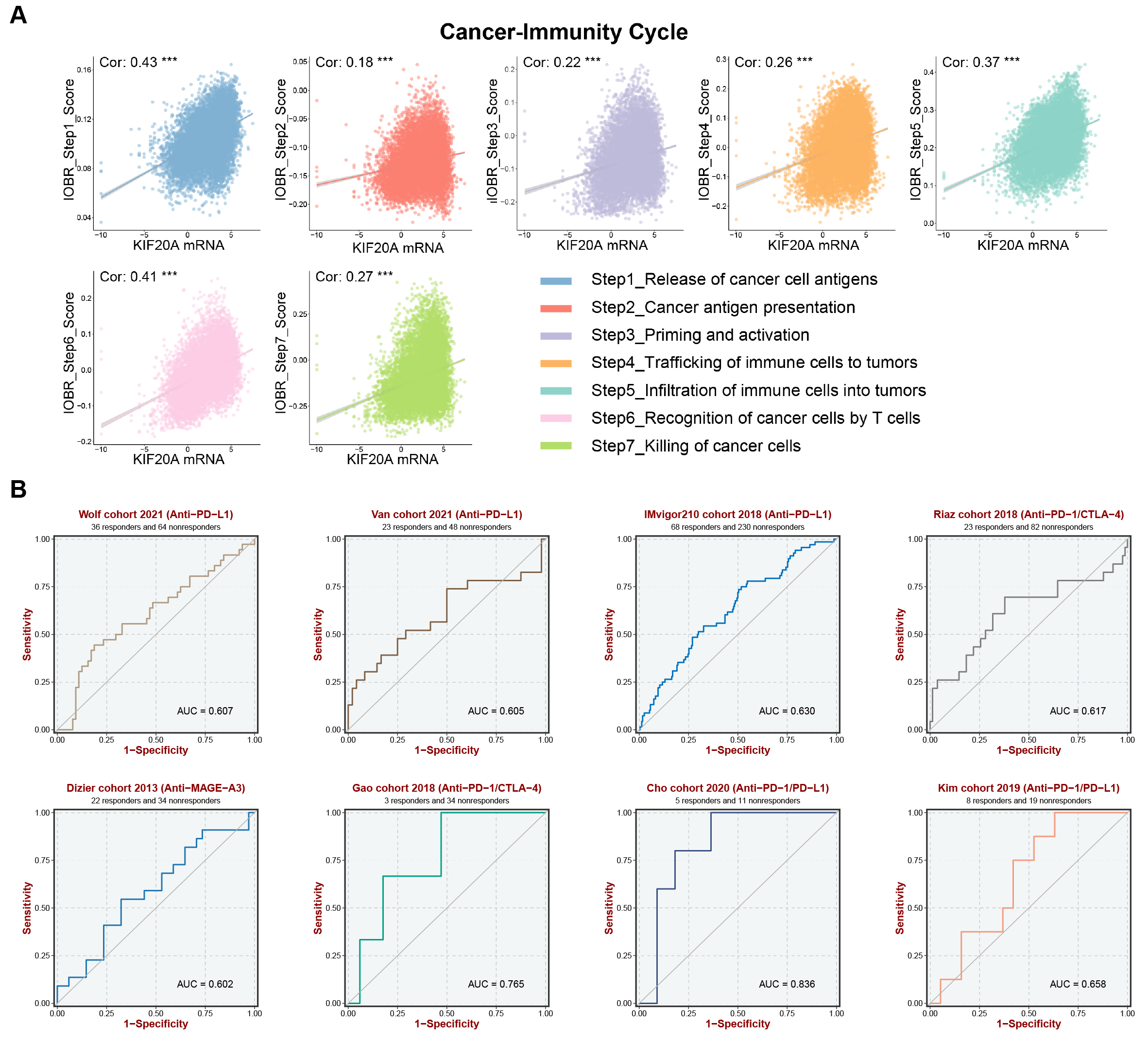

Supplement: Supplementary file 5 [file Image4.tif]

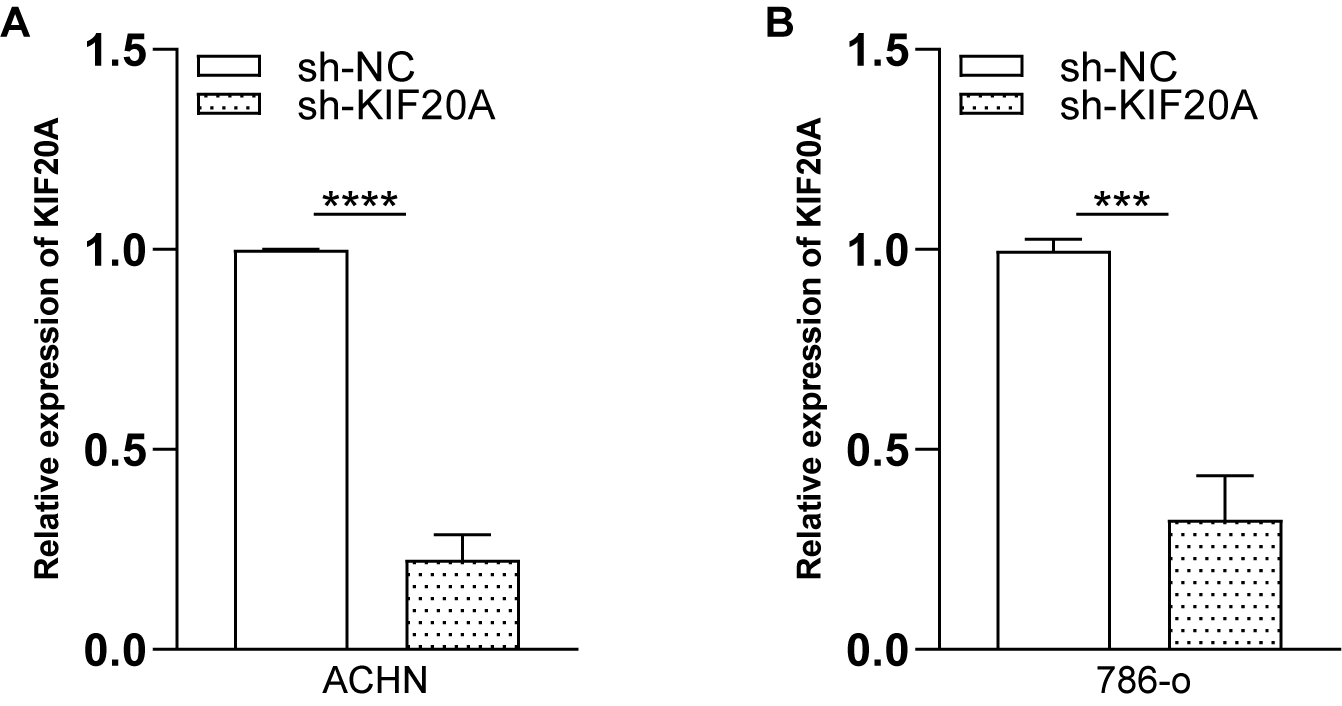

Supplement: Supplementary file 6 [file Image5.tif]
